# Evolutionary Analysis and Classification of OATs, OCTs, OCTNs, and Other SLC22 Transporters: Structure-Function Implications and Analysis of Sequence Motifs

**DOI:** 10.1371/journal.pone.0140569

**Published:** 2015-11-04

**Authors:** Christopher Zhu, Kabir B. Nigam, Rishabh C. Date, Kevin T. Bush, Stevan A. Springer, Milton H. Saier, Wei Wu, Sanjay K. Nigam

**Affiliations:** 1 Departments of Pediatrics, University of California at San Diego, La Jolla, California, United States of America; 2 Departments of Medicine, University of California at San Diego, La Jolla, California, United States of America; 3 Departments of Cellular and Molecular Medicine, University of California at San Diego, La Jolla, California, United States of America; 4 Departments of Molecular Biology, University of California at San Diego, La Jolla, California, United States of America; California State University Fullerton, UNITED STATES

## Abstract

The SLC22 family includes organic anion transporters (OATs), organic cation transporters (OCTs) and organic carnitine and zwitterion transporters (OCTNs). These are often referred to as drug transporters even though they interact with many endogenous metabolites and signaling molecules (Nigam, S.K., *Nature Reviews Drug Discovery*, **14**:29–44, 2015). Phylogenetic analysis of SLC22 supports the view that these transporters may have evolved over 450 million years ago. Many OAT members were found to appear after a major expansion of the SLC22 family in mammals, suggesting a physiological and/or toxicological role during the mammalian radiation. Putative SLC22 orthologs exist in worms, sea urchins, flies, and ciona. At least six groups of SLC22 exist. OATs and OCTs form two Major clades of SLC22, within which (apart from Oat and Oct subclades), there are also clear Oat-like, Octn, and Oct-related subclades, as well as a distantly related group we term “Oat-related” (which may have different functions). Based on available data, it is arguable whether SLC22A18, which is related to bacterial drug-proton antiporters, should be assigned to SLC22. Disease-causing mutations, single nucleotide polymorphisms (SNPs) and other functionally analyzed mutations in OAT1, OAT3, URAT1, OCT1, OCT2, OCTN1, and OCTN2 map to the first extracellular domain, the large central intracellular domain, and transmembrane domains 9 and 10. These regions are highly conserved within subclades, but not between subclades, and may be necessary for SLC22 transporter function and functional diversification. Our results not only link function to evolutionarily conserved motifs but indicate the need for a revised sub-classification of SLC22.

## Introduction

The solute carrier 22 protein family (SLC22 family) is a group of membrane transporters comprised of at least 31 known members in mouse and/or human [1]. The notion of the family became clear in 1997 with the cloning of what is now called OAT1 (originally NKT) [2,3]. At the time, the homology to OCT1 [4] and NLT (OAT2) [5] was noted, and a new transporter family was proposed [3]. Through cloning and genomic analysis in the past two decades, the family has since grown ten-fold. It is now well established that these transporters are involved in the transport of toxins, drugs and endogenous metabolites such as prostaglandins, urate, alpha-ketoglutarate, and carnitine across the cell membrane [1,6,7,8,9,10,11,12]. Members of this family have been classified in the IUBMB-approved Transporter Classification (TC) system within the Major Facilitator Superfamily (MFS) [13] (TC# 2.A.1) as organic anion transporters (OAT) and organic cation transporters (OCT) which denote the respective substrate preferences, although some substrates are shared by both [8,14]. In the last few years, there has been progress in understanding mammalian SLC22 proteins through site-specific mutagenesis of some transporters and construction of homology-based 3D models of SLC22A1 (OCT1), SLC22A2 (OCT2), and SLC22A6 (OAT1) [15,16,17,18].

The evolutionary basis of the SLC22 genes was described mostly prior to the complete genome sequencing of many relevant organisms; hence, the lineage of the SLC22 family remains somewhat unclear [7,19,20]. For example, when did the SLC22 proteins diverge into the various members in primates and rodents, and which are the ancestral members in each group? To best trace the lineage of the SLC22 transporters, specific organismal classes were chosen to represent the evolution of vertebrates and of the SLC22 family in parallel. Thus, we have analyzed SLC22 protein sequences from vertebrates including lamprey, shark, bony fish, amphibians, birds, monotremes, marsupials, mice, old/new world monkeys, and humans; invertebrate organisms such as fly (*Drosophilia melanogaster)*, worm (*Caenorhabditis elegans*), ciona (*Ciona intestinalis)*, and sea urchin (*Strongylocentrotus purpuratus)* were also included. Phylogenetic analyses indicate the origin of some SLC22 transporters that have clear orthologs in both humans and rodents which arose before the separation of bony fish and land vertebrates [21]. Six subclades were identified, including a relatively uncharacterized distant subclade consisting of SLC22A17, A18, A23, and A31; one of the members of this latter subclade (SLC22A18) may not be an authentic member of the SLC22 family, at least based on sequence analysis. Conserved motifs were analyzed in the context of the six subclades, and subclade-overrepresented motifs were defined. Available functional data on mutations in certain family members (OAT1, OAT3, OCT1, OCT2, OCTN2, URAT1) was evaluated in light of these motifs. In addition, there appears to be an expansion of SLC22 members, especially OATs, along with the evolution of mammals via gene duplication and divergence, supporting the potential importance of the SLC22 family in the development of mammals. Together, these analyses may prove useful for future studies to clarify the true physiological functions of family members in the various subclades of the SLC22 transporters, including their postulated role in remote communication (remote sensing and signaling). These analyses may also be helpful for identifying substrates of certain “orphan” members of the family [1,10,22,23,24].

## Materials and Methods

### Data

Mammalian SLC22 family protein sequences were collected using the NCBI BLASTp web interface with default parameters (the query sequence was the human ortholog of the appropriate sequence (hSLC22A1 for all SLC22A1). For the database option, a non-redundant protein sequence (nr) was chosen and no organisms were excluded. The algorithm chosen for all searches was the blastp option) [25]. Shark, lamprey, and sea urchin sequences were retrieved with BLASTp in each respective database: (http://esharkgenome.imcb.a-star.edu.sg/); (http://jlampreygenome.imcb.a-star.edu.sg/); (http://www.spbase.org/SpBase/).

### Phylogenetics

Sequences were aligned using ClustalX 2.1 (default parameters; Mode: Multiple Alignment Mode > Alignment > Do Complete Alignment) and/or the Multiple Alignment using Fast Fourier Transform (MAFFT, version 7) using default parameters [26,27]. Initially phylogenies were estimated using the neighbor-joining algorithm implemented in each of these programs. Sequence names and accession numbers are shown in S1 Table. The Interactive Tree of Life (http://itol.embl.de/) was used to display the phylogenies generated from ClustalX 2.1, and topology confidence was estimated with 1000 bootstrap replicates. MrBayes (http://mrbayes.sourceforge.net/) was run with default parameters on a MAFFT-aligned file of human and mouse sequences (only including human and mouse SLC22A1 through SLC22A31). The burn-in was set to 40000 generations to achieve a split frequency below 0.05 (This produced a topology similar to phylogenies produced by Clustal and MAFFT). This same analysis was repeated with sequences from sea urchin, fly, ciona, and shark along with available human SLC22 members (The resulting trees were also similar to those generated from ClustalX (S3 Fig)).

### Motif analysis

Motif comparisons were performed on the 31 SLC22 members included in this analysis using the Multiple Expectation-maximum for Motif Elicitation (MEME) suite (http://alternate.meme-suite.org/tools/meme) [28]. To detect any motifs found within the set of 111 SLC22 sequences, a threshold of 16 motifs at a range of 6 to 20 amino acid length was set with the normal discovery mode. This detection method yielded a set of evolutionarily conserved motifs across all members, SLC22A1 through SLC22A31 (S2 Table). A separate analysis using subclade-specific SLC22 members (Oat, Oct, Octn subclades) were then used at a threshold of 16 motifs at a range of 6 to 20 amino acid length in order to define the motif detection of the specific subclade. Motifs of several human homologs (SLC22A1-A6, A8, and A12) were then chosen from both the 111 sequence analysis (green) and subclade analysis (red); these were overlaid in a 2D representation of the proteins to further define residues overrepresented in a subclade. In the 2D representation of these sequences, the Web-based Hydropathy, Amphipathicity and Topology (WHAT) suite was used to predict transmembrane locations which use HMMTOP as the transmembrane prediction engine. Motif locations and transmembrane domain (TMD) locations were inputted into TOPO2 to draw the 2D representation (http://www.sacs.ucsf.edu/cgi-bin/open-topo2.py). This was repeated for Oat, Oct, Oat-like, Octn, Oct-related, and Oat-related subclades.

### Analysis of existing mutation data

Using the current literature available in the NCBI database (http://www.ncbi.nlm.nih.gov/), OAT1, OAT3, URAT1, OCT1, OCT2, OCTN1 and OCTN2 mutational data were collected. Functional changes in transport were noted for specific residues in each of the transporters mentioned (see S4 Table) and mapped onto their respective 2D topologies generated by TOPO2 (see S4–S6 Figs).

## Results

### Summary: phylogenetics of the SLC22 family members

**Fig 1:**
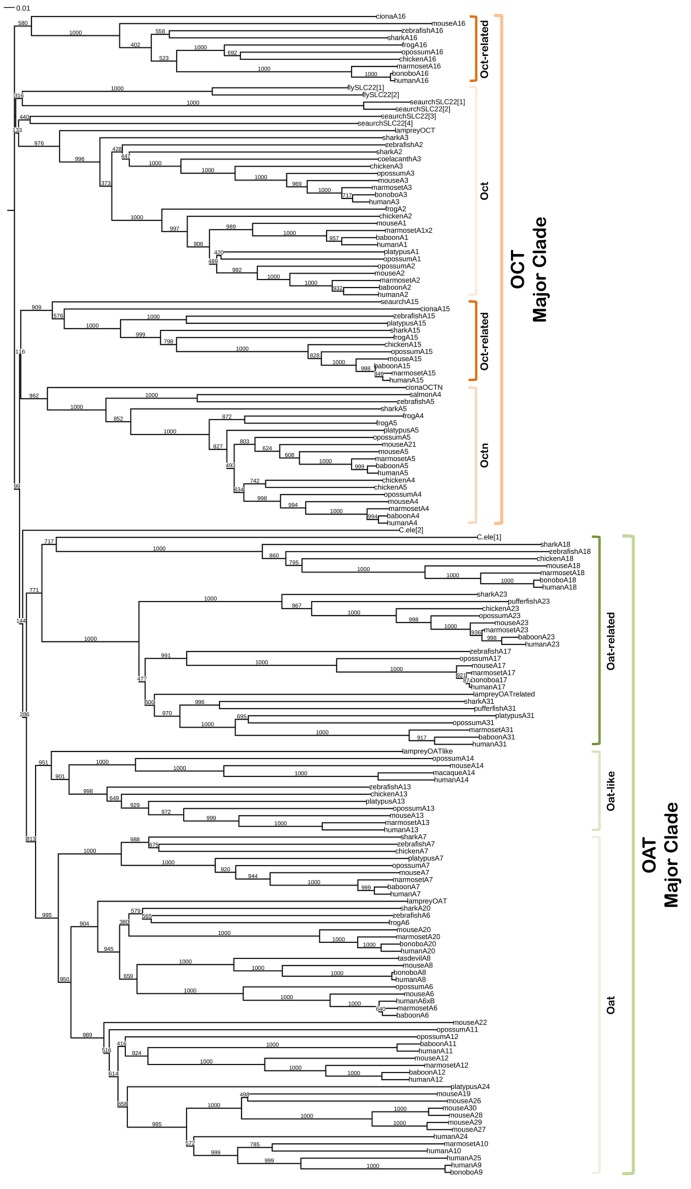
Unrooted Phylogeny of the SLC22 Transporter Family Using 163 Sequences of Various Organisms for Each of the 31 SLC22 Members. The sequences were aligned and the tree was generated using Clustal X 2.1 (using default parameters). The tree was viewed using Interactive Tree of Life (iTOL), an online tree viewing tool [55]. Accession numbers are listed in S1 Table. Bootstrap values are shown at each node of this phylogeny.

Our findings indicate that the SLC22 family members can be most clearly categorized into two major clades, the OAT Major clade and the OCT Major clade; within each of these Major clades, there exist several subclades. This leads to 6 groups that we have termed: Oat (to be distinguished from the OAT Major clade of which it is a subset), Oat-like, Oat-related, Oct (to be distinguished from the OCT Major clade of which it is a subset), Oct-related and Octn. Table 1 describes the various SLC22 members within each subclade. The OAT Major clade is colored orange with its subclades in various shades of orange. The OCT major clade is colored green with its subclades in various shades of green. S1 Table lists the sequence names and accession numbers used in the generation of Fig 1, S1 and S2 Figs. The organism classes were chosen based on their known evolutionary divergence from humans [21,29].

**Table 1:** List of Major and Sub Clades of SLC22 Family Members in Human and Mouse.

Major Clade	SubClade	Member (common name in human)	Human Chr. Location	Member (common name in mouse)	Mouse Chr. Location
OAT	Oat	SLC22A6 (hOAT1)	11q12.3	Slc22a6 (mOAT1)	19
OAT	Oat	SLC22A7 (hOAT2)	6p21.1	Slc22a7 (mOAT2)	17
OAT	Oat	SLC22A8 (hOAT3)	11q11	Slc22a8 (mOAT3)	19
OAT	Oat	SLC22A9 (hOAT7)	11q13.1		
OAT	Oat	SLC22A10 (hOAT5)	11q12.3		
OAT	Oat	SLC22A11 (hOAT4)	11q13.1		
OAT	Oat	SLC22A12 (hURAT1)	11q13.1	Slc22a12 (mURAT1)	19
OAT	Oat			Slc22a19 (Oat5)	19
OAT	Oat	SLC22A20 (hOAT6)	11q13.1	Slc22a20 (mOAT6)	19
OAT	Oat			Slc22a22 (Oatpg)	15
OAT	Oat	SLC22A24	11q12.3		
OAT	Oat	SLC22A25 (UST6)	11q12.3		
OAT	Oat			Slc22a26	19
OAT	Oat			Slc22a27	19
OAT	Oat			Slc22a28	19
OAT	Oat			Slc22a29	19
OAT	Oat			Slc22a30	19
OAT	Oat-like	SLC22A13 (hOAT10)	3p21.3	Slc22a13 (mOAT10)	9
OAT	Oat-like	SLC22A14 (hOCTL2)	3p21.3	Slc22a14 (mOCTL2)	9
OAT	Oat-related	SLC22A17 (hBOCT1)	14q11.2	Slc22a17 (mBOCT1)	14
OAT	Oat-related	SLC22A18 (hORCTL2)	11p15.5	Slc22a18 (mORCTL2)	7
OAT	Oat-related	SLC22A23 (C6orf85)	6p25.2	Slc22a23 (3110004L20Rik)	13
OAT	Oat-related	SLC22A31	16q24.3		
OCT	Oct	SLC22A1 (hOCT1)	6q25.3	Slc22a1 (mOCT1)	17
OCT	Oct	SLC22A2 (hOCT2)	6q25.3	Slc22a2 (mOCT2)	17
OCT	Oct	SLC22A3 (hOCT3)	6q25.3	Slc22a3 (mOCT3)	17
OCT	Octn	SLC22A4 (hOCTN1)	5q31.1	Slc22a4 (mOCTN1)	11
OCT	Octn	SLC22A5 (hOCTN2)	5q23.3	Slc22a5 (mOCTN2)	11
OCT	Octn			Slc22a21 (Octn3)	11
OCT	Oct-related	SLC22A15 (hFLIPT1)	1p13.1	Slc22a15 (mFLIPT1)	3
OCT	Oct-related	SLC22A16 (hFLIPT2)	6q22.1	Slc22a16 (mFLIPT2)	10

### Orthologs and paralogs among SLC22 transporters

In an effort to provide novel insights into SLC22 transporter functions, we sought to identify orthologous and paralogous subclades. When two genes are orthologs, their phylogenetic topology matches the branching order that prevails for the majority of the genome: Their differences have evolved via speciation events (i.e. mouse *Slc22a2* versus human *SLC22A2*). Lamprey has SLC22 family sequences that are orthologs of each Oat, Oct and Oat-like subclade (Fig 1). Paralogs can originate by gene duplication (i.e. human *SLC22A1* vs human *SLC22A2*) [30]. In humans there are 23 SLC22 genes, at least 1 of which appears unique to primates (*SLC22A25*). In rodents there are 25 SLC22 genes, 5 of which appear unique to rodent SLC22 transporters (*Slc22a26*-*Slc22a30*) that were recently described [20]. From our database searches, we have also found 16 SLC22 genes in marsupials, 8 SLC22 genes in monotremes, 10 SLC22 genes in birds (chicken), 6 SLC22 genes in amphibians (frog), 13 SLC22 genes in bony fish (fugu, salmon, zebrafish), 10 SLC22 genes in shark, and possibly 4 SLC22 genes in lamprey (S1 Table). Distant SLC22 orthologs have been previously reported in fly, worm and other species, but it is often difficult to determine to which subclade they belong (Oat, Oct or Octn) [7].

### Major clades and subclades

Phylogenies identified several subclades within the two Major clades of OAT and OCT. Fig 1 describes the general overview of aligned proteins from all members used in this analysis (SLC22A1 –SLC22A31). The supplementary figures (S1 and S2 Figs) provide greater evolutionary detail of the SLC22 family members within each subclade. To differentiate the grouping patterns within a phylogenetic tree, the term clade was chosen to describe the rooted estimation of the phylogenies for the SLC22 amino acid sequences. The first split between the OAT and OCT classes of SLC22 were defined as the Major OAT and Major OCT clades. The subsequent subclades within the Major clades were grouped according to known identification. For genomic grouping, the term “cluster” was chosen to describe the chromosomal locations for each clade if the members were located next to each other on a chromosome.

### The general overview

Fig 1 was generated using the results from NCBI’s BLASTp [25] from 163 known SLC22 family members A1 through A31 (S1 Table). MAFFT and ClustalX alignments are in agreement that there appear to be two major branches which then split into various minor branches. Bayesian reconstructions return similar overall topologies (S3 Fig). The larger branch of the two is the Organic Anion Transporter (OAT) Major clade consisting of three subclades which include the Oat subclade (SLC22A6-A12, A19-A22, A24-A30), Oat-like subclade (A13, A14), and an “Oat-related” subclade (A17, A18, A23, A31). The Oat subclade is by far the best taxonomically represented subclade, especially within mammals, which includes SLC22A6-SLC2A8 (OAT1, OAT2, OAT3). Within this subclade, there are also species-specific members such as SLC22A25 (UST6) for primates [20] (S1 Fig). The Oat-like subclade consists of members SLC22A13 and SLC22A14 (OAT10 and OCTL2) (S1 Fig).

Many of these proteins (OATs, OCTs, OCTNs) have been shown, by in vitro transport assays and to some extent in vivo knockouts, to transport organic anions, cations and zwitterions. However, the nomenclature can sometimes be confusing; for example, organic anion transporters (OATs) can transport certain cationic substrates, despite being largely organic anion transporters in vitro and in vivo [10,14,31,32,33]. The Oat-related subclade consists of members SLC22A17, A18, A23, and A31 (S1 Fig). The evolutionary distance suggests that the “Oat-related” subclade is structurally and functionally unique, although still related enough by sequence to be considered with the prototypical OAT and zwitterionic subclades as opposed to the Oct subclade (further discussed below). Although not well studied, current literature suggests they may have different substrates and different expression patterns than the OATs and OCTs [34,35].

A second distinct branch has been identified as the Organic Cation Transporter (OCT) Major clade. The OCTs further branch into three more closely related subclades: the classical organic cation transporters (Oct) subclade (SLC22A1-A3) (S2 Fig), and the Octn and Oct-related subclades (SLC22A4-A5 and SLC22A15-16), respectively (S2 Fig). As presented, SLC22A1-A3 genes are found in many different vertebrates including humans, birds, and bony fish. Although the exact functions of the orthologs may vary from organism to organism, SLC22A1-A3 are known to transport many cationic drugs and metabolites, while SLC22A4-5 (OCTN1-2) and SLC22A15-16 (FLIPT1 and FLIPT2 originally identified in 2002) [36] are generally considered zwitterionic transporters with carnitine as a potential physiological substrate [20].

Because there were several branches with exceedingly low bootstrap values, specifically regarding the invertebrate species [1,7], a separate alignment was generated (data not shown) to confirm that the putative SLC22 members for fly, worm, and sea urchin belonged to the Major OAT or OCT clade. The resulting alignment placed homologs from these lower species within the OCT Major clade.

### OAT and OCT major clades

In order to view the OAT and OCT Major clades without mutual alignment influences from each other, the OAT and OCT Major clades were also separately analyzed (S1 and S2 Figs). Also, additional sequences were included within the subclades (e.g. Oat-like, Octn) to further define the evolutionary details.

#### Oat subclade

S1 Fig uses 175 sequences from NCBI. The Oat subclade contains 16 known members including, SLC22A6, SLC22A7, SLC22A8, SLC22A9, SLC22A10, SLC22A11, SLC22A12, SLC22A20, SLC22A22, SLC22A24, SLC22A25, and SLC22A26—SLC22A30. The prototypical OAT, OAT1 (SLC22A6), was first identified as NKT [3]. As presented here, SLC22A6 or OAT1 is found in nearly all classes of vertebrate organisms included in this analysis with the exception of birds and monotremes. In the Japanese lamprey genome database, a protein match was found and included in this phylogeny (jlampreygenome accession JL188). Interestingly, this sequence appears equally related to SLC22A6 and its close relative, SLC22A20, which is highly expressed in olfactory mucosa in mammals (S1 Fig) [37,38,39]. Although a shark Slc22a6 was not found, the presence of the shark Slc22a20 relative, along with the evidence of fish SLC22A6 orthologs in zebrafish and salmon, support ancestry in this subclade. SLC22A8, another close relative of SLC22A6, may have first appeared in marsupials. SLC22A7 appears to be as equally ancestral as SLC22A6 and is represented in nearly all classes of vertebrate organisms used in this analysis including cartilaginous fish, bony fish, birds, rodents, marsupials, and placental animals. These analyses place the development of the ancestral OAT3 gene after the development of the OAT1 and OAT2 genes; however, additional analysis will be required to determine whether OAT1 or OAT2 is most ancestral. The several Slc22a6 sequences belonging to various fish appear to align best with SLC22A20, also known as OAT6 in mice [38]. This may suggest that OAT1 (SLC22A6) and OAT6 (SLC22A20) evolved in parallel with each other or that these fish sequences are more similar to the mammalian SLC22A20 than expected. Sequence identity analysis shows that zebrafish SLC22A6 is 49% identical to both mouse Slc22a6 and Slc22a20.

Interestingly, there appears to be a mammalian expansion of SLC22 members consisting of the members SLC22A9, SLC22A10, SLC22A11, SLC22A12, SLC22A22, SLC22A24, SLC22A25, and SLC22A26-SLC22A30. BLASTp searches suggest SLC22A25 to be unique to primates and SLC22A26-A30, which are part of a single genomic cluster, to be unique to rodents [20]. This expansion of SLC22 members appears unique to mammals and was found only within the Oat subclade.

#### Oat-like subclade (SLC22A13-A14)

SLC22A13 (OAT10) is a urate transporter expressed in the kidney and to some extent, the brain [40]. Our analyses support an ancestry that, in this subclade, extends to lamprey (accession JL188). SLC22A13 has also been found in zebrafish, chicken, platypus, and all mammals used in this analysis. SLC22A14 appears to be more evolutionarily recent with its first appearance in opossum. This subclade appears to be a close relative of the larger Oat subclade (S1 Fig).

#### Oat-related subclade (SLC22A17, A18, A23 and A31)

The Oat-related subclade is in a separate branch in this phylogeny. This subclade is a somewhat closer relative of the traditional organic anion transporters (OATs and OAT-like) than the organic cation transporters (OCTs) based on the branching schemes calculated (Fig 1). Both MAFFT and neighbor-joining alignments support the view that SLC22 members A17, A18, A23, and A31 appear to group together. Although these four group together in the general overview (Fig 1), there appears to be a subdivision within this group between A18 and A17, A23, and A31. SLC22A17 is shown to exist in organisms, up to opossum, and there is possibly a related sequence in lamprey (jlampreygenome accession JL10482). SLC22A23 and SLC22A31 were found to exist in all mammals as well as the bony fishes such as pufferfish and coelacanth, although it is possible these fish sequences have functionally diverged. Interestingly, the *C*. *elegans* (NCBI accession AAF73198.1) sequence (identified as a putative OAT sequence in 2004 based on homology to fly-like transporters or FLIPT) [7] was found to align within this subclade, though it is unclear which member of this subclade is most similar to it in multiple alignments. SLC22A18 appears somewhat diverged compared to the rest of the Oat-related subclade. However, bootstrap support is high enough to conclude that these four SLC22 members are the most distantly related subclade of the SLC22 family (S1 Fig).

Because of the sequence divergence seen in this subclade relative to the other SLC22 members, other drug transporter families were investigated for homology. Members of the Drug H^+^ Antiporter-1 (DHA1, TC# 2.A.1.2) family, best described in bacteria, yeast and fungi, were found to have high similarity to SLC22A18 in separate alignments. The top hits with TCBLAST scores of at least 100 were included in a multiple global alignment of SLC22 family members A1-A31 (data not shown). The findings suggest SLC22A18 may be more similar to DHA1 sequences than those of the SLC22 family. This implies that SLC22A18 is more distantly related to the SLC22 family than previously suggested and perhaps is related to the DHA1 family. Further studies to determine whether SLC22A18 is truly a member of this subclade, and possibly the SLC22 family itself, are warranted.

#### Oct subclade (SLC22A1-A3)

134 sequences were used to generate S2 Fig. The Oct subclade consists of members SLC22A1, SLC22A2, and SLC22A3. A BLASTp search of the NCBI database found the Oct subclade (SLC22A1, SLC22A2, and SLC22A3) to be found in a wide range of organisms. From the Japanese lamprey genome database, an uncharacterized protein with close similarity to the Oct subclade was found, implying ancestry in this family to at least the evolution of lampreys (jlampreygenome accession JL2643) and sharks (S2 Fig). As is apparent from the available sequences, SLC22A1 (OCT1) is present only in mammals, while SLC22A2 (OCT2) and SLC22A3 (OCT3) ancestry extends to lampreys [41].

#### Octn and Oct-related subclade (SLC22A4, A5, A15, A16 and A21)

Alignments indicate SLC22A4 (OCTN1) and SLC22A5 (OCTN2) to be within the Major OCT clade (Fig 1). The OCTNs have been previously described as carnitine transporters in mice and humans [42,43]. Both SLC22A4 (OCTN1) and SLC22A5 (OCTN2) are present in marsupials (opossum), monotremes (platypus), avians (chicken), bony fish (zebrafish and salmon), ciona, and sea urchin implying ancestry in this group before the separation of bony fish and land animals over 450 million years ago [21,44,45]. Slc22a21 (Octn3) is found to exist only in mice and is closely related to existing rodent Slc22a5 (S2 Fig).

SLC22A15 and SLC22A16, previously described as FLIPT1 and FLIPT2, are related to the Oct and Octn subclades by sequence [36]. SLC22A16 (FLIPT2) also known as CT2, transports carnitine. It is thought to be somehow involved in the development of spermatozoa in humans [46]. The “Oct-related” subclade members (SLC22A15 and SLC22A16) do not group very well with the Oct or Octn subclades, nor do they group very well relative to each other (Fig 1). The lineage of the human variants SLC22A15 and SLC22A16 therefore appeared to have diverged substantially from the better known Oct and Octn subclades. Additional work will be required to further distinguish when these members diverged from the rest of the Oct and Octn subclades or if these are a separate category of SLC22 members.

### Ancestral subclade predictions

The Oct, Oat, and Oat-like subclades are similar in phylogenetic topology, and each includes one lamprey homolog. The Oat-related (SLC22A17, A18, A23, A31) subclade appears to have both a lamprey homolog, as well as a putative worm SLC22 member. This suggests that the Oat-related subclade may predate the Oat, Oct, and Oat-like subclades. However, there were no fly or sea urchin homologs found in either NCBI or their genome-specific databases that grouped with the Oat-related subclade. The Oct-related subclade (FLIPT) appears to diverge substantially from the other subclades of the OCT major clade. However, several sea urchin sequences appear to belong to the Oct-related subclade with one sea urchin sequence (NCBI accession XP_003730263.1) annotated as SLC22A15 (FLIPT1).

### Invertebrates within the phylogeny

Putative worm, fly, sea urchin, and ciona SLC22 sequences are included in this phylogenetic analysis. The topology of the branches indicates that the invertebrate sequences align in the Octn or Oct-related subclade (FLIPT1 and FLIPT2 or SLC22A15 and A16). However, the bootstrap support for the exact subclade to which they belong is low. Based on the available data, Fig 1, indicates the OCT Major clade predates the OAT Major clade. With the exception of a single *C*. *elegans* sequence in the OAT major cluster, only the OCT Major clade contains the invertebrate organisms such as fly, worm, and sea urchin. Despite the ambiguity among the invertebrate sequences, several of them appear to have high enough bootstrap values to group with one of the existing subclades. Ciona sequences appear in the Octn, SLC22A15, and SLC22A16 (Oct-related) subclades (NCBI accession numbers XP_002132109.1, XP_009860851.1, XP_002124941.1, respectively). A single sea urchin sequence was found to belong in the SLC22A15 subclade (NCIB accession number XP_003730263.1). These findings indicate that much of the OCT Major clade originated during or before the split between the OCT and OAT Major clades.

Of the SLC22 fly and worm sequences previously identified, 4 worm and 9 fly sequences were inputted into the global phylogeny [7]. Based on the phylogeny indicated there, these invertebrate ‘homologs’ are equally distantly related to the Oct and Octn subclades.

### Subclade specific motifs

**Fig 2:**
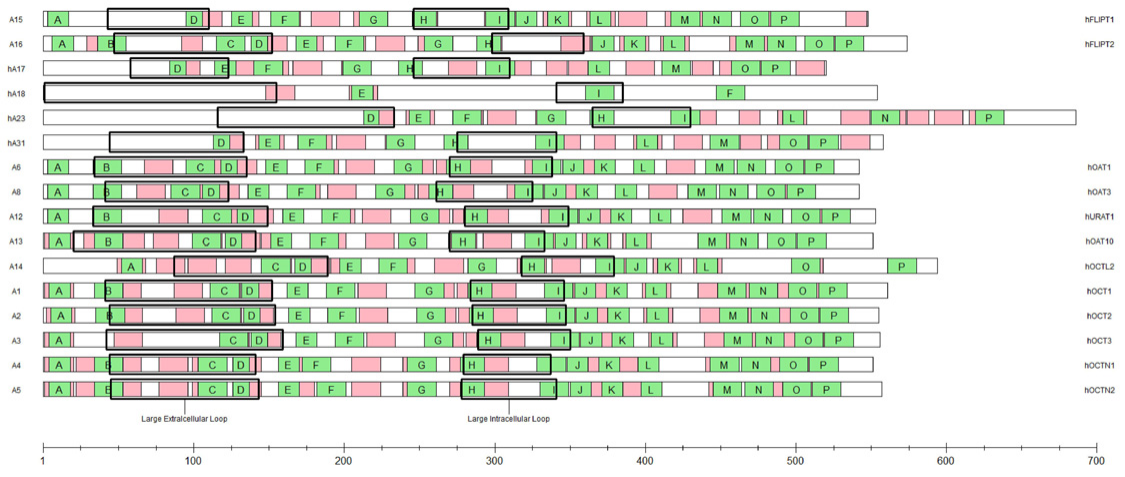
Shared Motifs of SLC22 Family Transporters by MEME Analysis on 16 of the Sequences Used in the Analysis. Abbreviations of the SLC22 member number is on the far left (e.g., A15 = SLC22A15) and known common names are on the far right. In green are the SLC22 family-conserved motifs which are lettered (A-P) in the order that they appear in the SLC22A6 amino acid sequence. The majority of the sequences follow this motif order. In red are the subclade-specific motifs overlaid on the same 1D representation of each SLC22 sequence. The first box labeled the Large Extracellular Loop represents the predicted Large Extracellular Loop [56] and the second box labeled the Large Intracellular Loop represents the predicted Large Intracellular Loop. Note that each 1D representation is proportionally scaled such that each sequence length can be estimated in the scale near the bottom of the figure.

Prototypical SLC22 members used as reference sequences for motifs are as follows (Fig 2): Oat subclade includes SLC22A6, SLC22A8, and SLC22A12 (hOAT1—NP_695008.1, hOAT3—NP_004245.2, hURAT1—NP_653186.2) with 88 sequences inputted into MEME. The Oat-like subclade includes SLC22A14 (hOCT-like2—NP_004794.2) with 22 sequences inputted into MEME. The Oct subclade includes SLC22A1, SLC22A2, and SLC22A3 (hOCT1—NP_003048.1, hOCT2—NP_003049.2, hOCT3—NP_068812.1) with 51 sequences inputted into MEME. The Octn subclade includes SLC22A4 and SLC22A5 (hOCTN1—NP_003050.2, hOCTN2—NP_003051.1) with 38 sequences inputted into MEME. The Oct-related subclade includes SLC22A15 and SLC22A16 (hFLIPT1—NP_060890.2 and hFLIPT2—NP_149116.2) with 35 sequences inputted into MEME. The subclade motifs were compared to a set of 16 motifs of 111 sequences including SLC22 members A1-A31. All motifs found had significant E-values of less than 0.05. Supplemental S2 Table contains 16 motif sequences from each of the members above. Subclade MEME inputted sequences and accession numbers can be found in S5 Table. The Oat-related subclade MEME analysis did not produce many significant overlapping motifs. Topology predictions for the Oat-related members also appeared less reliable and are not displayed.

The members of the Oat subclade contain a unique area within the first extracellular region beginning on L67 in SLC22A6 (hOAT1). Homologous sections in the other two members, SLC22A8 (hOAT3) and SLC22A12 (hURAT1), also show this substantial 20 amino acid length motif. Other significant motifs include an H130-M142, L253-F259, and a region L283-R298 on hOAT1 and homologous sections in hOAT3 and hURAT1.

The Oat-like members SLC22A13 and SLC22A14 contain motif topologies similar to the Oat subclade members. There exists a subclade-specific 18 amino acid motif in both SLC22A13 (P73-P90) and SLC22A14 (P121-P137). Both members also contain homologous motifs T214-F233 (SLC22A13) and Q292-V311 (SLC22A14).

Within the Oct subclade, an interesting finding emerges when comparing the three OCT members: SLC22A1, SLC22A2, and SLC22A3 (OCT1, OCT2, and OCT3). SLC22A1 and SLC22A2 share a homologous motif (G87-T106 in OCT1) which excludes SLC22A3. This suggests SLC22A1 and SLC22A2 to be sister genes while SLC22A3 to be a sister gene of an ancestral SLC22A1/A2.

The Octn subclade shows a similar motif location beginning on V77-G96 on both SLC22A4 and SLC22A5, OCTN1 and OCTN2 respectively. Surprisingly, there is a lack of a motif beginning at the ‘ESPXR’ region found in many other SLC22 transporters in this family.

Multiple alignments show the Oct-related subclade (FLIPT) is the most distant of the OCT Major clade members. SLC22A15 (FLIPT1) does not share several common domains that are expected in the SLC22 transporter family in the first extracellular loop. This difference in extracellular domain could be a significant factor in its characterization as an SLC22 transporter and could imply differences in function relative to the other SLC22 family members. SLC22A16 (FLIPT2) contains the common motifs found in the first extracellular region as well as a member-defining motif, N92-R106. Similar to the Octn subclade, the Oct-related subclade lacks a significant motif found in the better known SLC22 family members such as SLC22A1 and SLC22A2 in the ‘ESPXR’ domain.

### Mapping of existing disease mutations, SNP (single nucleotide polymorphisms) and mutagenesis data

Drug transporters are of considerable medical, physiological, pharmaceutical and toxicological importance (reviewed in [1,10]). Among SLC22 transporters, for example, single nucleotide polymorphisms (SNPs) in OAT1 (SLC22A6), OAT3 (SLC22A8), OCT1 (SLC22A1) and OCT2 (SLC22A2) have been implicated in altered drug and toxin responses. URAT1 (SLC22A12) appears to be one of the major uric acid transporters in the kidney, and mutations and SNPs in it have been associated with altered uric acid levels, gout and kidney stones. OCTN2 (SLC22A5) is a carnitine transporter, and mutations are associated with systemic carnitine deficiency. As a result of these clinical implications, a great deal of functional transport data exists for these transporters (S4 Table). We sought to overlay these data on the various conserved motifs to analyze their potential functional relevance.

**Fig 3:**
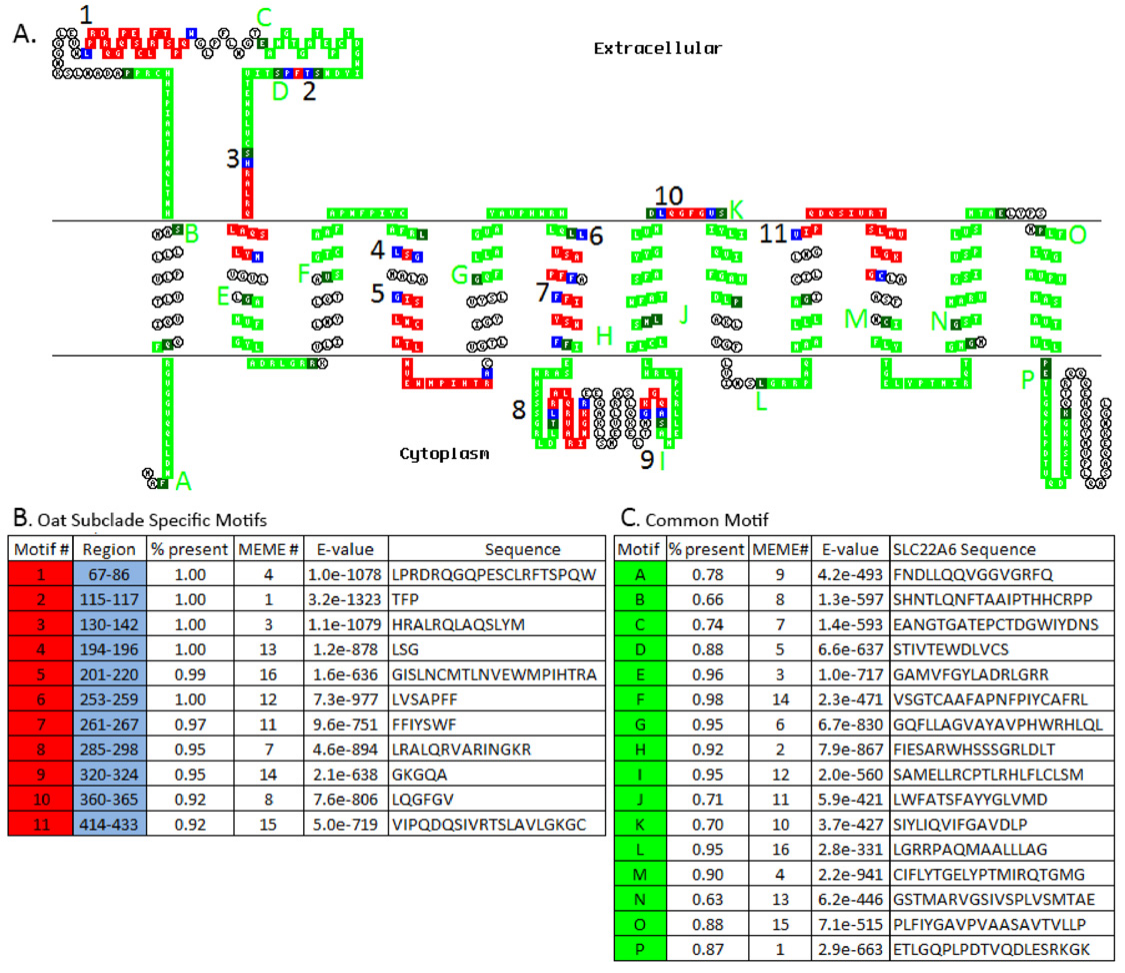
Overlay of Common and Subclade-specific Motifs on Human SLC22A6 2D Topology. (A) SLC22 family-conserved motifs versus subclade-conserved motifs. Overlaid on the 2D topology are the 16 evolutionarily-conserved family motifs (green/dark green) and the 11 subclade-specific motifs (red/blue). Conserved family motifs are assigned letters A-P in the order of appearance in human OAT1 (human SLC22A6). Subclade-specific motifs are either whole or partial (red/blue) and are assigned the numbers 1–11. (B) This table describes the order, region, and percent present of the motifs which appear in the inputted set, MEME#, E-value, and amino acid sequence of each of the subclade-specific motifs identified for the hOAT1 sequence (red). (C) This table describes the SLC22 family-conserved motifs that have been overlaid onto the hOAT1 sequence (green). It lists the motif letter and percent present in all 111 sequences used in the MEME analysis, the MEME#, E-value, and the amino acid sequence of hOAT1 associated with the particular motif letter. Additional sequence information can be found in S3 and S4 Tables.

Mutagenesis and SNP data were gathered and mapped onto their respective 2D topologies of OAT1, OAT3, URAT1, OCT1, OCT2, and OCTN1/2. OCTN1 and OCTN2 data were mapped onto the OCTN2 2D model because of the relatively high identity and similarity between them and because much of the data available were of OCTN2 origin (S6 Fig, S3 Table). Three areas of interest emerged from the mapping: the first large extracellular loop, the central region near or at the large intracellular loop, and the 9^th^ and 10^th^ transmembrane domains. Interestingly, many of the mapped residues that were found in the first large extracellular loop reside in the clade-specific motif identified in this study (Fig 3; colored in red). The other residues that appear crucial for transport reside in TMD 9 and 10 which have been confirmed in various uptake experiments. Another interesting observation is that SNPs associated with disease, in the case of URAT1 and OCTN2, fall near or within the signature ‘ESPXR’ region of these proteins, the central intracellular loop of the SLC22 family transporters.

### Oat-related subclade

The Oat-related subclade, consisting of the remaining SLC22 members A17, A18, A23, and A31, contains 10 of 16 motifs detected in the A1-A31 analysis. Unlike the 5 other subclades identified, the Oat-related subclade does not contain a clear member that can be generalized for the other members. In the motif detection within all 31 members, the human SLC22A17 sequence only contains 9 out of the 16 common motifs detected; human SLC22A18 sequence contains 3 out of the 16 motifs commonly detected; human SLC22A23 contains 9 out of the 16 common motifs; human SLC22A31 contains 10 of the 16 common motifs. Within the clade-specific MEME motif detection, the SLC22A17 sequence contains the most motifs in common with the subclade at 16 of 16 subclade motifs detected, followed by SLC22A31 (15/16), SLC22A23 (14/16), and SLC22A18 (2/16). The relatively few common motifs found within SLC22A18 suggest it is the most distantly related member of the SLC22 family. It will be important to obtain functional data to determine whether its membership in SLC22 is justified.

### Gene locations

Pairing and clustering of the SLC22 family members in human and rodent genomes have been previously described [20,47]. Using the UCSC genome browser and previously mapped gene locations for the SLC22 genes, gene clusters in human were identified. The majority of the Oat subclade exists on chromosome 11. The Oat-like subclade, SLC22A13 and SLC22A14, exists on chromosome 3. The Oct subclade, SLC22A1, A2, and A3, clusters on chromosome 6. The Octn subclade clusters on chromosome 5. However, there are several human SLC22 members within these subclades that do not follow the genomic pattern. SLC22A7 (OAT) and SLC22A23 (Oat-related) are found on chromosome 6, which the OCT cluster is also on, although not part of the same phylogenetic clade. SLC22A15 (hFLIPT1) appears on chromosome 1. SLC22A17 and SLC22A31, both part of the Oat-related subclade, also exist on separate chromosomes without other known SLC22 members nearby on chromosome 14 and chromosome 16, respectively. SLC22A18 (Oat-related) exists on chromosome 11 on which the known OAT cluster exists 60 million base pairs away.

## Discussion

Our phylogenetic analysis of 31 mammalian SLC22 transporters, as well as non-mammalian, species indicates the existence of 6 subclades, including an evolutionary distant and functionally unclear subgroup consisting of SLC22A17, A18, A23, and A31. Using the available genome databases along with the current NCBI database, putative ancestral genes were also identified within the subclades. Furthermore, by defining motifs overrepresented in different subclades and analyzing the considerable functional data related to disease mutations, functional SNPs, and experimental mutagenesis, the functional importance of various evolutionarily conserved motifs in some subclades was also highlighted.

### Expansion of SLC22 proteins in mammals

An interesting finding that emerges from the phylogenetic analysis is that there was an expansion of certain SLC22 transporter subclades in mammals through gene duplication and divergence from which SLC22A1, SLC22A8, SLC22A9, SLC22A10, SLC22A11, SLC22A12, SLC22A14, SLC22A18, SLC22A19, SLC22A21, SLC22A22, and SLC22A24-SLC22A30 have evolved [20]. This suggests a role for these SLC22 genes in the basic physiology of mammals and possibly in their successful radiation during the last 60 million years. The majority of the observed expansion appears to be among organic anion transporters (OATs).

Organic cation transporters (OCTs) and organic carnitine/zwitterion transporters (OCTNs) expansion is limited to SLC22A1-5 and SLC22A15-A16. OCT1 (SLC22A1) appears to be mammalian-exclusive (S2 Fig). Current literature on the functionality of each of the cation/zwitterion subclades (Oct, Octn, and Oct-related) includes the transport of drugs, metabolites and toxins for OCTs and OCTNs; there is also a possible role in the maturation of spermatozoa in humans for the “OCT-related” genes FLIPT1 and FLIPT2 (SLC22A15-A16) [46].

The “Oat-related” cluster contains members SLC22A17, A18, A23, and A31. SLC22A17 and SLC22A23 reportedly do not share many of the common OCT substrates associated with the SLC22 family such as 1-methyl-4 phenyl-pyridinium (MPP+) and carnitine despite showing expression in the choroid plexus and liver, similar to many of the known SLC22 members [34,35]. There is also evidence that SLC22A18, an outlier in many respects (as described above) may play a role in the development of non-small cell lung cancer [48].

### Non-mammalian SLC22 proteins

Fish SLC22 genes have diverged substantially from their mammalian orthologs, which seems reasonable from an evolutionary standpoint because fish and mammals have different physiological requirements and encounter different small molecules in their respective environments. OCT2 has duplicated genes in salmon and tetraodon, which is consistent with genome duplication in teleosts [49,50]. Of note, alignments within our analysis show zebrafish slc22a6 grouped with known mouse and human SLC22A20 orthologs, which suggests some ambiguity as to which subclade certain fish orthologs belong.

An interesting finding was that BLAST searches did not find certain SLC22 members in frogs, platypuses or opossums. Their absence could be due to loss of the gene or to incomplete sequencing of the genome. Of note is that the platypus is missing several genes that would be expected to be present. For example, platypus lacks an ortholog of SLC22A2 (OCT2), which does not seem consistent with the presence of SLC22A2 (OCT2) in birds and fish (S2 Fig). However, platypus contains an ortholog of SLC22A3 (OCT3), which may perform some of the functions of SLC22A2.

Lamprey and shark sequences were identified which have similarity with known SLC22 subclades. These subclades include the Oct (SLC22A1-A3), the Oat, the Oat-like subclades (SLC22A13-A14), and the Oat-related subclade. Based on our analyses, the OAT cluster appears the most ubiquitous throughout all organisms. SLC22A6 (OAT1) is the best known within this cluster and is expressed in the kidney and other tissues [31]. Furthermore, transport capabilities of OAT1 and others within this cluster (OAT2—OAT10) have been documented and these transporters are found to have affinities for hundreds of endogenous and exogenous compounds that are mostly, though not exclusively, organic anions of considerable physiological, as well as toxicological and pharmaceutical significance [10,24,51,52].

Ancestry of the SLC22 genes appears to extend as far as the worm; however, bootstrap support for some of the lower branches remains low (Fig 1). It appears OCTs predate OATs in evolutionary time because of the invertebrate SLC22-like transporters (see Oct-related subclade, S2 Fig) that exist in ciona, worms, flies, and sea urchins. These findings imply a basic function for the SLC22 transporters that have not been fully characterized and should be investigated for further clarification regarding their true physiological functions [1]. The most distantly related of the SLC22 family, the Oat-related subclade, is most likely a unique subset of the SLC22 family. However, current sequencing data indicate that it has existed as long as many of the better known SLC22 members and that it is possibly even older.

### Motifs found within the SLC22 family

The motif patterns found within our subclades place a particular focus on the first extracellular domain with several TMDs uniquely present in some cases. This is particularly clearly seen when comparing 2D topologies of SLC22A1, SLC22A6, and SLC22A12, all of which show subclade-specific domains in this region as well as the flanking common motifs. The evolutionarily conserved flanking regions may hold the structurally important features of the SLC22 family while the subclade-specific motif region may allow for the transporter’s flexibility in handling different sets of substrates. SNPs in SLC22A6 (OAT1), SLC22A8 (OAT3), and SLC22A12 (URAT1) have suggested stabilizing selection in this extracellular region, which implies the sequence and structure of this region may be important for its function [53]. Mutagenesis studies of SLC22A6 and SLC22A8 revealed that cysteine residues on TMD 10 are related to the sensitivities to Hg^2+^, a region that was detected in our analyses to be a significant motif within the Oat subclade [54].

Because of the tremendous clinical importance of various SLC22 transporters (OAT1, OAT3, OCT1, OCT2, URAT1, OCTN2) in pharmacology and disease (e.g. hyperuricemia, systemic carnitine deficiency), a great deal of functional analysis of mutations and SNPs already exists (reviewed in [1], S4 Table). This enabled us to analyze functionally relevant mutations in the context of evolutionarily conserved motifs in the subclades. Indeed, certain mutagenesis studies that might have been proposed based on our evolutionary analysis of motifs have already been performed, although, to our knowledge, they have not been systemically analyzed from this viewpoint.

The motif predictions along with mutagenesis and SNP data suggests there are 3 conserved areas of these proteins that are responsible for function; the first extracellular domain, the central large intracellular domain, and the adjacent transmembrane domains 9 and 10. The substrate kinetics associated with the reported residues suggests these three domains work in conjunction with one another to help govern substrate specificity as no single residue or domain appears solely responsible for transport (S4–S6 Figs). Additional information per residue can be found in S4 Table.

### Genomic locations

Genomic locations and the relative similarities of these genes strongly suggest duplication events which have produced the SLC22 subclades we have found [20,49]. The motif analyses and available substrate data also suggest initial functional similarities between the duplicated sister genes that have diverged over time into the various subclades present. Some SLC22 family members do not cluster on the same locations as do the Oat, Oct, and Octn subclades. Thus, other factors may be at play that explain the distribution of the SLC22 family members across different chromosomes. Nevertheless, the close similarity of these clustered sequences and the similarity in substrates for current members of the same clade indicate there might have been shared ancestrally similar functions between the SLC22 members; for example in the Oat subclade, all homologs with substrate data presumably classified as organic “anion” transporters also cluster together on chromosome 11 with the exception of SLC22A7 (OAT2).

In summary, we have defined subclade-selective motifs (S4–S6 Figs) and analyzed their relationships to function, based upon disease mutations, functionally important SNPs, and experimental mutagenesis data (S4 Table). We present evidence for SLC22 transporters in worms, flies, ciona and sea urchin. However, there appears to have been a major expansion in mammals, presumably related to physiological and toxicological requirements. As described in the Remote Sensing and Signaling hypothesis, “drug” transporters are likely to play important roles in homeostatic physiology, injury-repair, and development [1,10,22,24]. Mammals, in particular, may have taken advantage of the endogenous functions of the organic anion transporters (OATs) and developed a mammalian-exclusive SLC22 family member set (SLC22 members A6-A14 and A19-A31). Additional work on the Oat-related members, which are currently not functionally well-characterized, may hold important clues as to how these members have diverged in function while maintaining sequence similarity. Motif analysis suggests that the first extracellular region is important for function in each particular subclade as it is highly conserved across members of a subclade (Oat/Oct/Octn) while not conserved across members of different subclades. The conserved regions across all SLC22 members appear to flank the subclade-specific regions which include extracellular, cytoplasmic, and transmembrane regions. By mapping on to these motifs the functional data related to specific residues important in substrate transport, we were able to support the view that three distinct regions of SLC22 transporters (first extracellular domain, large intracellular domain, and TMD 9/10) function together to mediate the particular transport capabilities of subclade members.

## Supporting Information

S1 Fig The OAT Major Clade. 175 sequences were used to generate this figure. The branching scheme outlined here shows the three subclades we have identified and is rooted on the most ancient sequence, *C*. *elegans* (C.ele[1]). Three subclades were identified: the Oat subclade (to be distinguished from the OAT Major clade), the Oat-like subclade, and the Oat-related subclade. Bootstrap values in this phylogeny describe the confidence in each node. The naming of each leaf reflects the common name of the species that was used as well as the isoform number (denoted after the “x”), if available. S1 Table lists the accession numbers used to generate this phylogeny.
(PDF)

S2 Fig The OCT Major Clade. This figure was generated from 133 sequences from various organisms. Three subclades were identified: the Oct subclade (to be distinguished from the OCT Major clade), the Octn subclade, and the Oct-related subclade. The phylogeny has been rooted to the most ancient sequence, *C*. *elegans*. Invertebrate sequences are difficult to assign to subclades due to their low bootstrap values within this phylogeny, as well as their relatively low similarity to the other members of the OCT Major clade. The naming of each leaf reflects the common name of the species that was used as well as the isoform number if available (indicated after the “x”). S1 Table lists the accession numbers used to generate this phylogeny.
(PDF)

S3 Fig MrBayes Generated Tree. A phylogenetic tree generated using the MrBayes software using the aligned sequences converted into nexus format.
(PDF)

S4 Fig Mapped OAT Residues. Mutagenesis and SNP data collected for OAT1, OAT3, and URAT1. Human variants were chosen to display these residues; other model organism variant data are starred. Residues for which mutagenesis or SNP data exist are indicated directly on the topology. (Green, residues common to all SLC22s; red/blue, residues found in subclade-specific motifs).
(PDF)

S5 Fig Mapped OCT Residues. This series of figures describes mutagenesis and SNP data collected for OCT1 and OCT2. As in Supplemental S4 Fig, mutated residues are displayed as human variants, while any other model organism variant data have been starred. Mutagenesis and SNP data are indicated directly on the topology. (Green, residues common to all SLC22s; red/blue, residues found in subclade-specific motifs).
(PDF)

S6 Fig Mapped OCTN Residues. Mutagenesis and SNP data were analyzed for OCTN1 and OCTN2. Human OCTN2 was chosen to represent the residues found because the majority of the available data was of hOCTN2 origin. OCTN1 residues are also mapped (starred) on this sequence because sequence similarity between OCTN1 and OCTN2 is relatively high. Residues for which mutagenesis and SNP data are available are indicated directly on the topology. (Green, residues common to all SLC22s; red/blue, residues found in subclade-specific motifs).
(PDF)

S1 Table List of Sequences and Accession Numbers for Generating Fig 1, Supplemental S1 and S2 Figs. (PDF)

S2 Table Evolutionarily Conserved Motif Sequences. (PDF)

S3 Table Clade Specific Sequences. (PDF)

S4 Table Specific Residue Information. (PDF)

S5 Table MEME Input. (PDF)
