# Supplementary figures and images for: Evolutionary Analysis and Classification of OATs, OCTs, OCTNs, and Other SLC22 Transporters: Structure-Function Implications and Analysis of Sequence Motifs
Source: PLoS One. 2015 Nov 4;10(11):e0140569. doi: 10.1371/journal.pone.0140569 (PMC4633038; doi:10.1371/journal.pone.0140569)

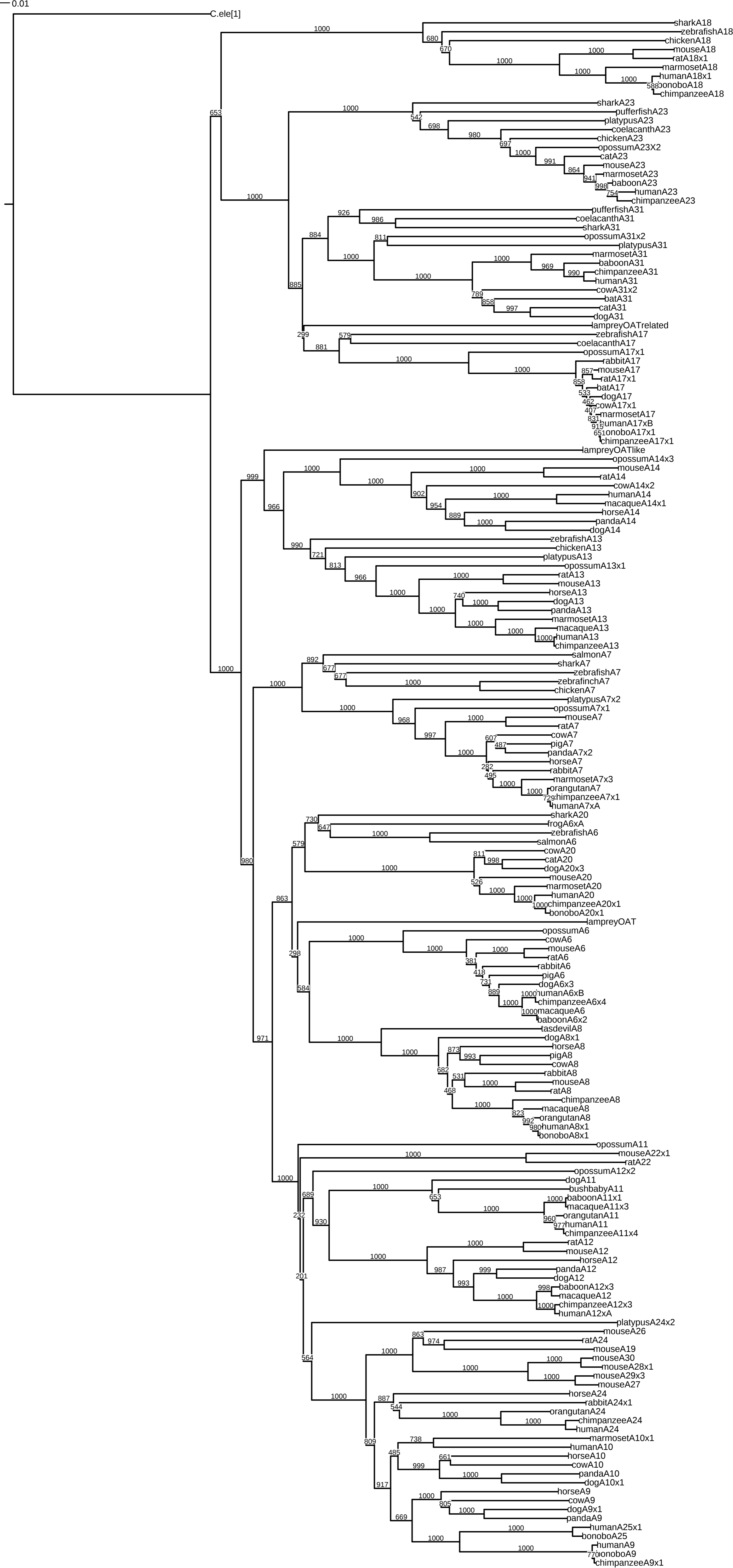

Supplement: S1 Fig — 175 sequences were used to generate this figure. The branching scheme outlined here shows the three subclades we have identified and is rooted on the most ancient sequence, C. elegans (C.ele[1]). Three subclades were identified: the Oat subclade (to be distinguished from the OAT Major clade), the Oat-like subclade, and the Oat-related subclade. Bootstrap values in this phylogeny describe the confidence in each node. The naming of each leaf reflects the common name of the species that was used as well as the isoform number (denoted after the “x”), if available. S1 Table lists the accession numbers used to generate this phylogeny. (PDF) [file pone.0140569.s001.pdf]

— 0.01

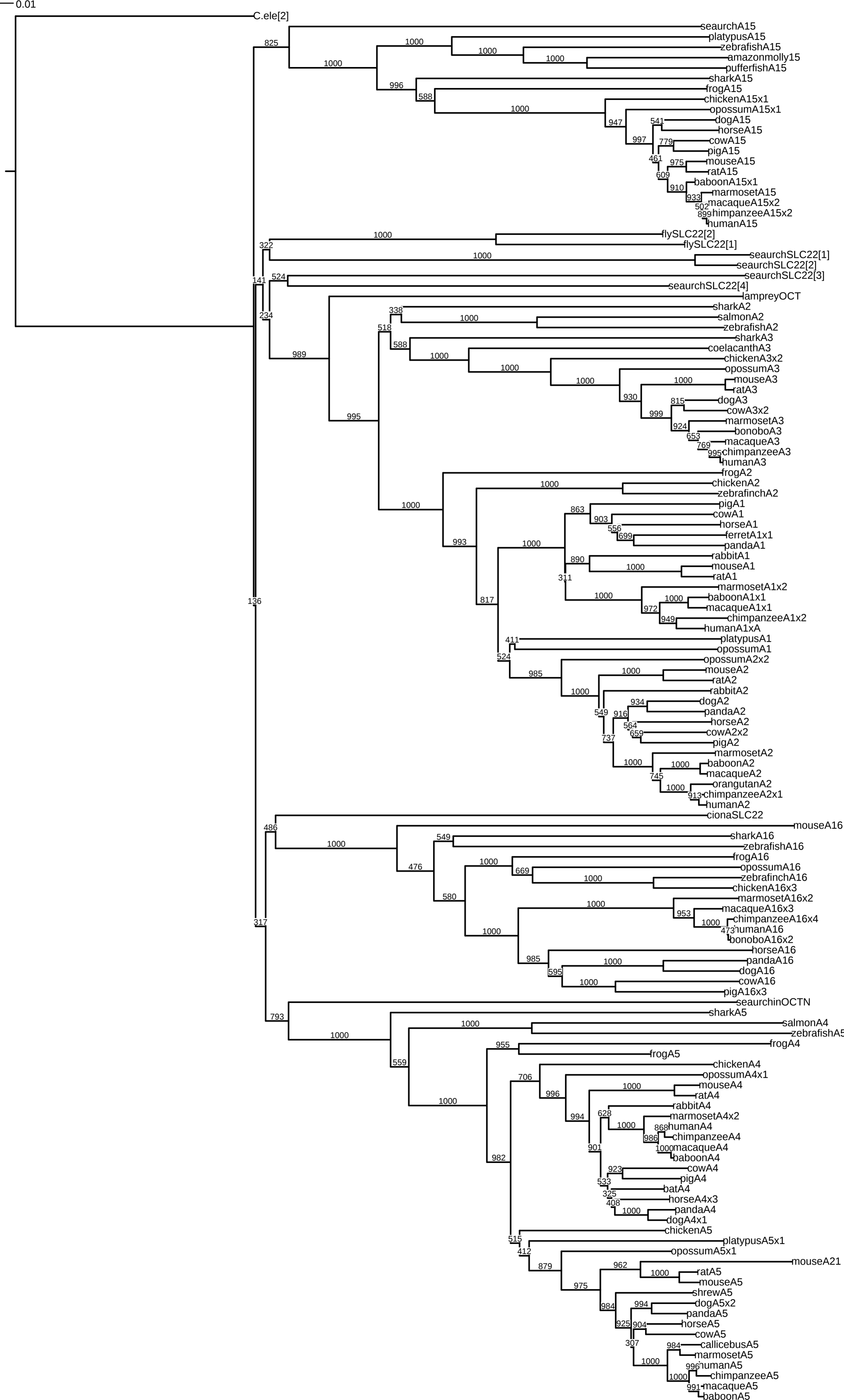

Supplement: S2 Fig — This figure was generated from 133 sequences from various organisms. Three subclades were identified: the Oct subclade (to be distinguished from the OCT Major clade), the Octn subclade, and the Oct-related subclade. The phylogeny has been rooted to the most ancient sequence, C. elegans. Invertebrate sequences are difficult to assign to subclades due to their low bootstrap values within this phylogeny, as well as their relatively low similarity to the other members of the OCT Major clade. The naming of each leaf reflects the common name of the species that was used as well as the isoform number if available (indicated after the “x”). S1 Table lists the accession numbers used to generate this phylogeny. (PDF) [file pone.0140569.s002.pdf]

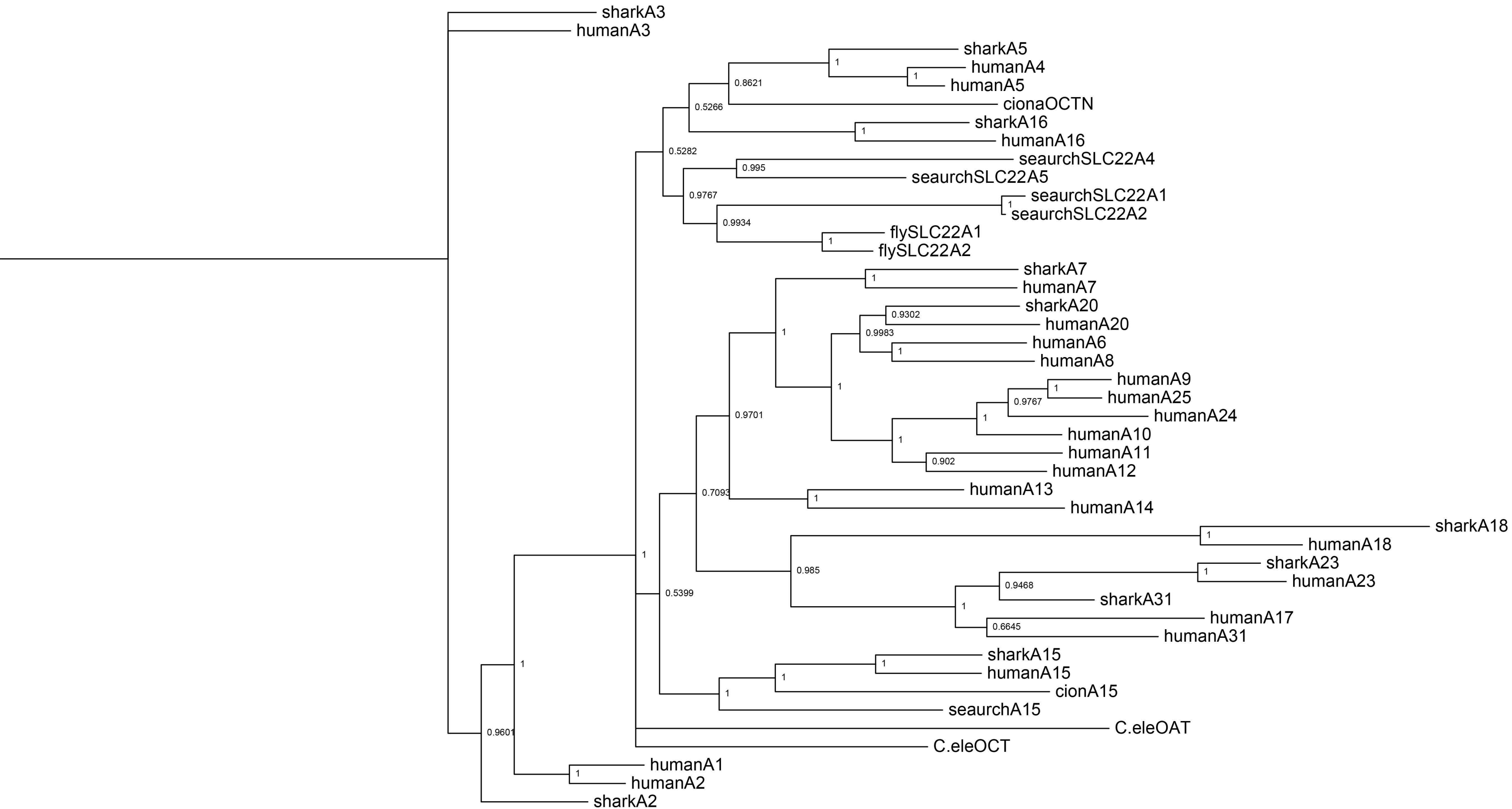

Supplement: S3 Fig — A phylogenetic tree generated using the MrBayes software using the aligned sequences converted into nexus format. (PDF) [file pone.0140569.s003.pdf]

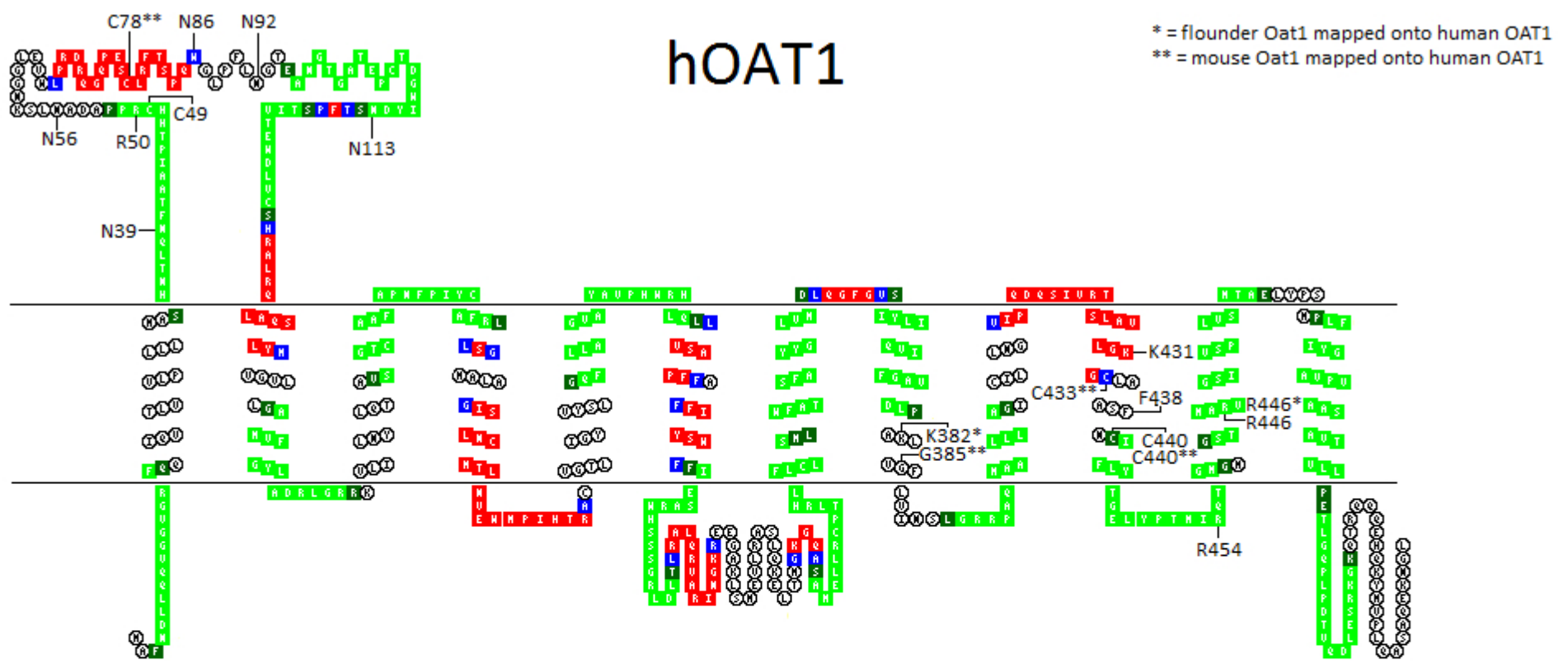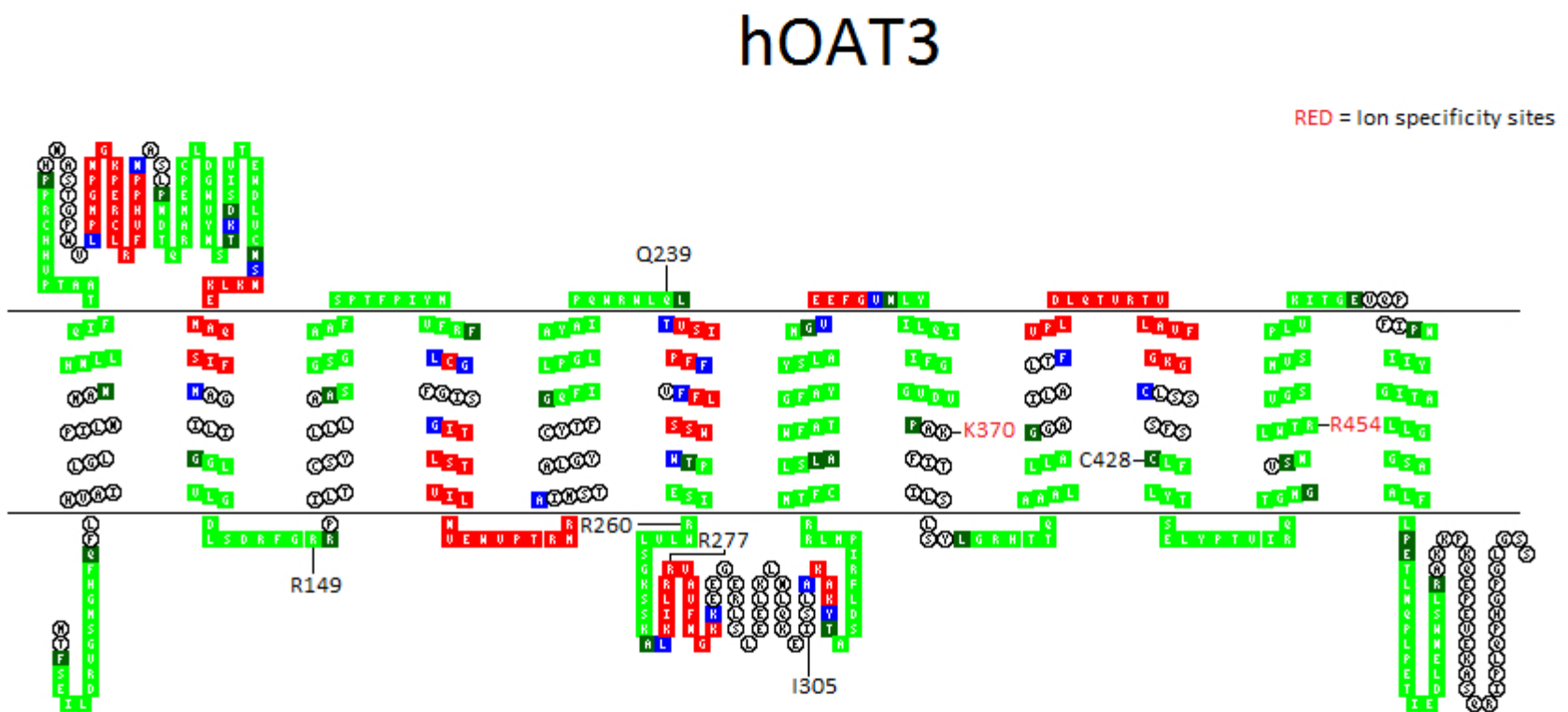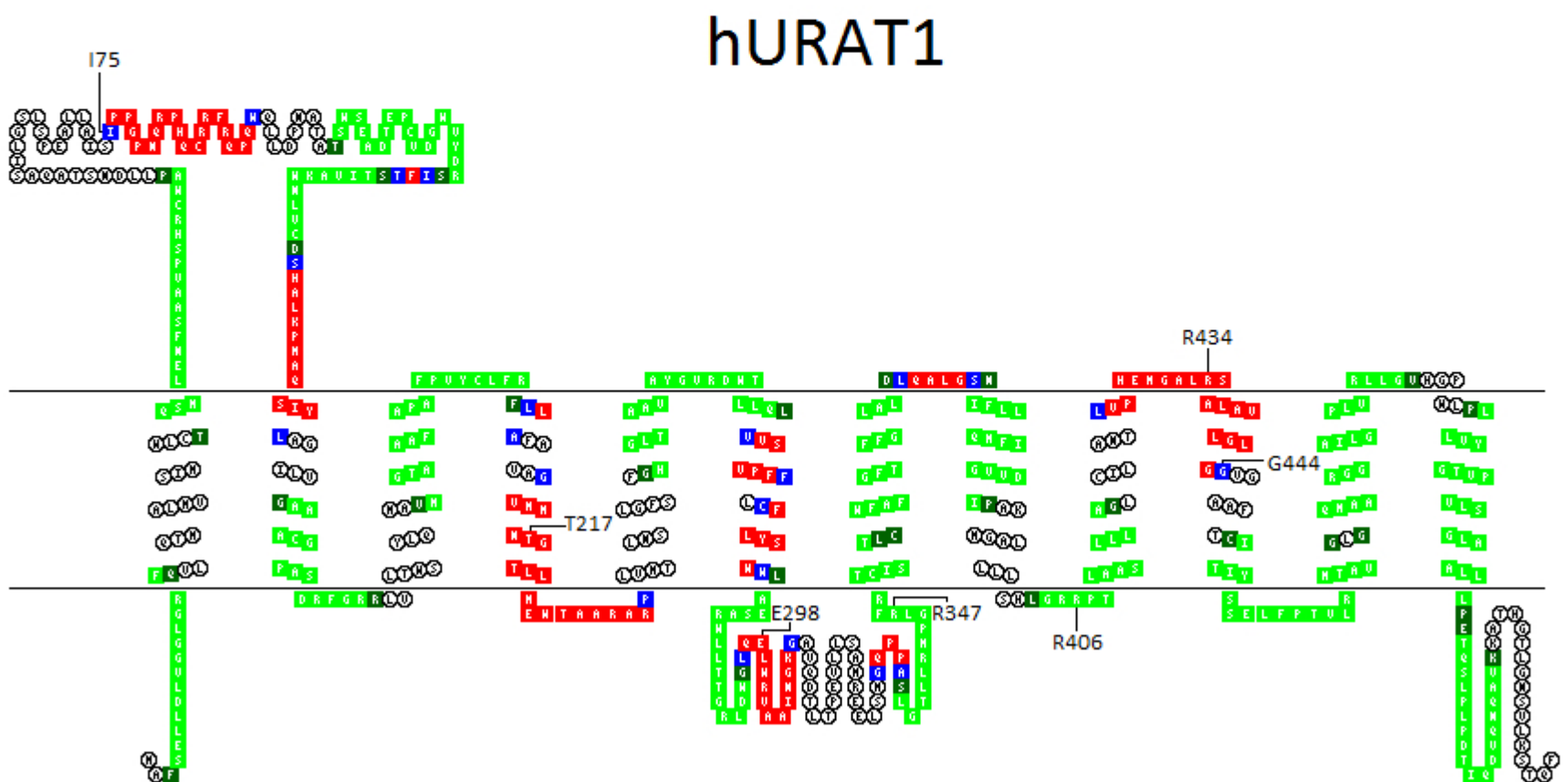

Supplement: S4 Fig — Mutagenesis and SNP data collected for OAT1, OAT3, and URAT1. Human variants were chosen to display these residues; other model organism variant data are starred. Residues for which mutagenesis or SNP data exist are indicated directly on the topology. (Green, residues common to all SLC22s; red/blue, residues found in subclade-specific motifs). (PDF) [file pone.0140569.s004.pdf]

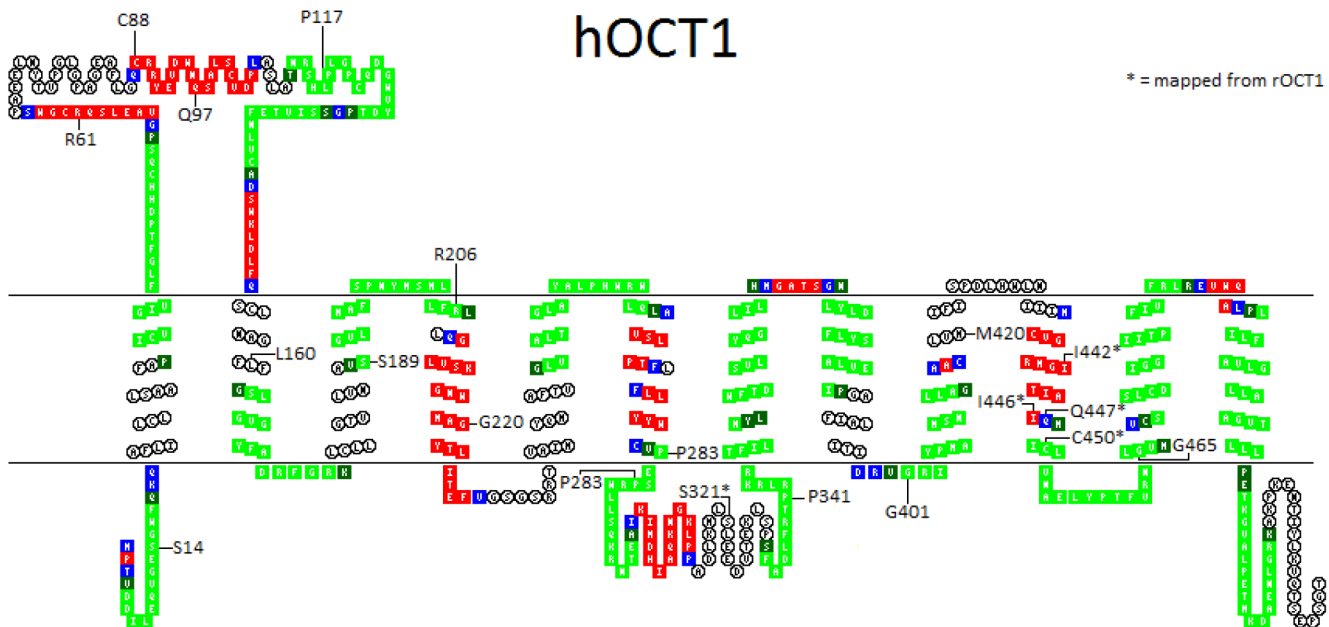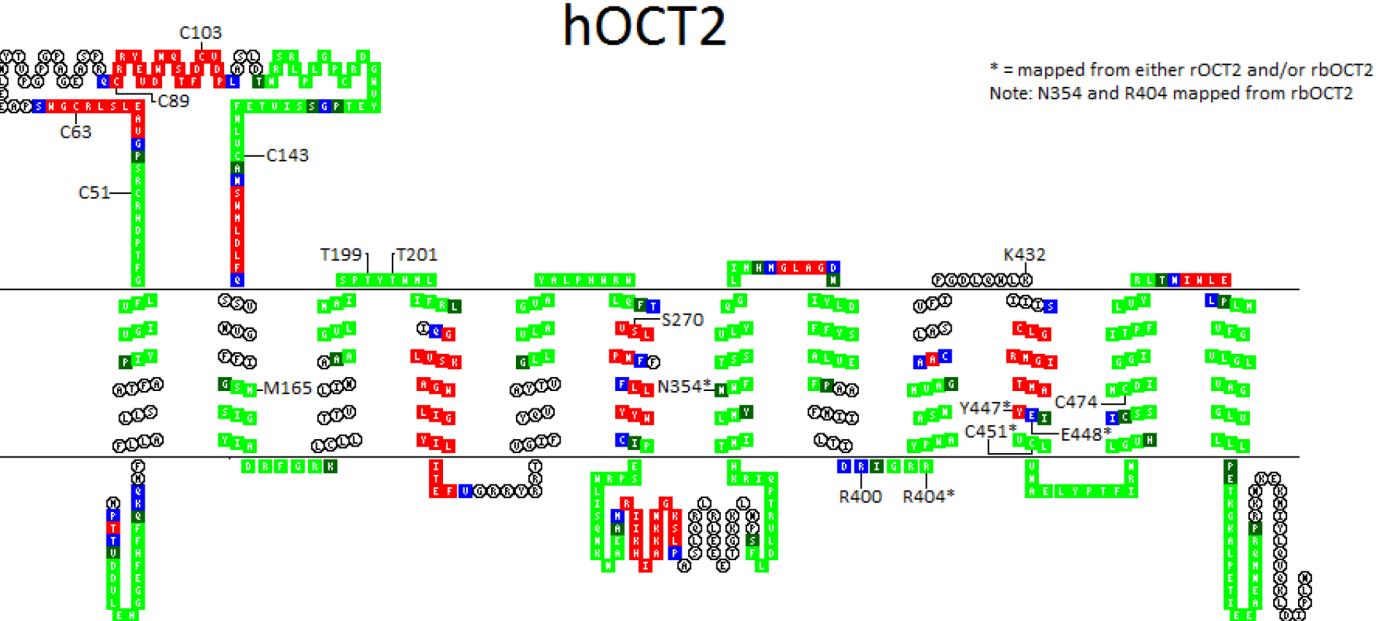

Supplement: S5 Fig — This series of figures describes mutagenesis and SNP data collected for OCT1 and OCT2. As in Supplemental S4 Fig, mutated residues are displayed as human variants, while any other model organism variant data have been starred. Mutagenesis and SNP data are indicated directly on the topology. (Green, residues common to all SLC22s; red/blue, residues found in subclade-specific motifs). (PDF) [file pone.0140569.s005.pdf]

## hOCTN2

\* = mapped from OCTN1

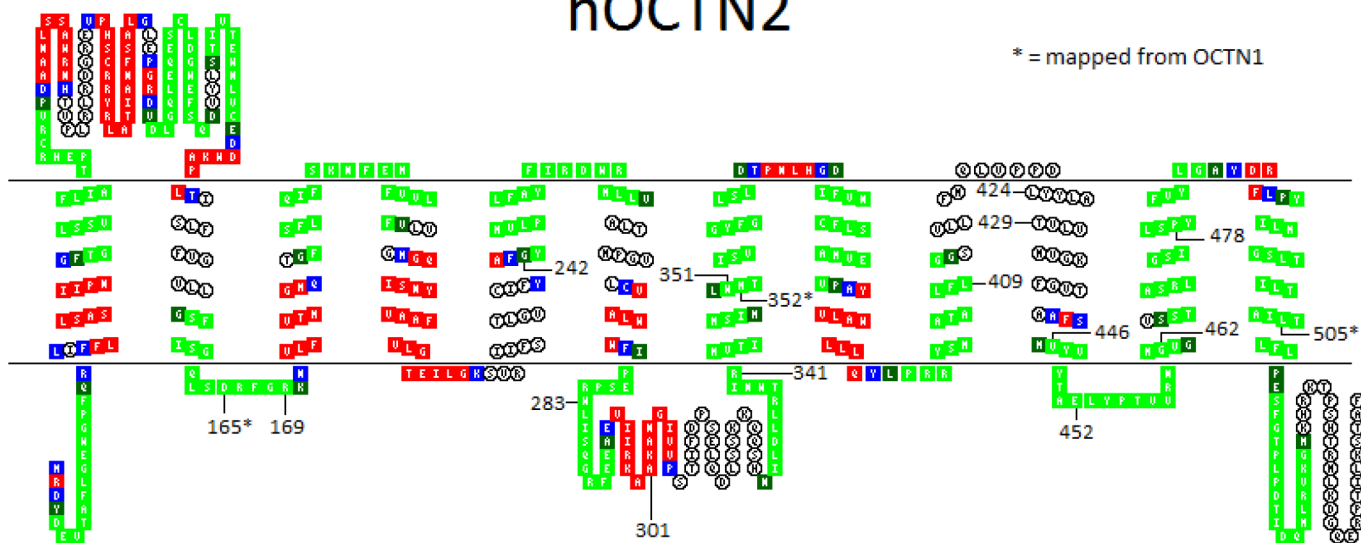

Supplement: S6 Fig — Mutagenesis and SNP data were analyzed for OCTN1 and OCTN2. Human OCTN2 was chosen to represent the residues found because the majority of the available data was of hOCTN2 origin. OCTN1 residues are also mapped (starred) on this sequence because sequence similarity between OCTN1 and OCTN2 is relatively high. Residues for which mutagenesis and SNP data are available are indicated directly on the topology. (Green, residues common to all SLC22s; red/blue, residues found in subclade-specific motifs). (PDF) [file pone.0140569.s006.pdf]
