# Supplementary material for: Evolutionary Analysis and Classification of OATs, OCTs, OCTNs, and Other SLC22 Transporters: Structure-Function Implications and Analysis of Sequence Motifs
Source: PLoS One. 2015 Nov 4;10(11):e0140569. doi: 10.1371/journal.pone.0140569 (PMC4633038; doi:10.1371/journal.pone.0140569)
Supplement: S1 Table — (PDF) [file pone.0140569.s007.pdf]

Supplemental Table 1
List of sequence names and accession numbers for Figures 1, S1, and S2

Sequence Name	Accession Number	Figure 1	Figure S1	Figure S2
amazonmolly15	XP_007568088.1			X
baboonA1	XP_003898408.1	X		X
baboonA11	XP_003909652.1	X	X	
baboonA12	XP_003909651.1	X	X	
baboonA15	XP_003892505.1	X		X
baboonA2	XP_003898410.1	X		X
baboonA23	XP_003897025.1	X	X	
baboonA31	XP_003917369.1	X	X	
baboonA4	NP_001162226.1	X		X
baboonA5	NP_001162227.1	X		X
baboonA6	XP_003909713.1	X	X	
baboonA7	XP_003897681.1	X		
batA17	XP_006776882.1		X	
batA31	XP_005862737.1		X	
batA4	ACC68852.1			X
bonoboA16	XP_008976203.1	X		X
bonoboa17	XP_003808141.1	X	X	
bonoboA18	XP_003816095.1	X	X	
bonoboA20	XP_003828666.2	X	X	
bonoboA25	XP_003828572.1		X	
bonoboA3	XP_003807452.1	X		X
bonoboA8	XP_003828567.1	X	X	
bonoboA9	XP_003828570.1	X	X	
bushbabyA11	ACH92547.1		X	
C.ele[1]	AAF73198.1	X	X	
C.ele[2]	Q9U539.3	X		X
callicebusA5	ACB21281.1			X
catA20	XP_003993638.1		X	
catA23	XP_006931564.1		X	
catA31	XP_004001564.1		X	
chickenA13	XP_418529.3	X	X	
chickenA15	XP_416558.2	X		X
chickenA16	XP_419787.3	X		X
chickenA18	XP_421021.3	X	X	
chickenA2	XP_419622.2	X		X
chickenA23	XP_418968.3	X	X	
chickenA3	XP_419620.4	X		X
chickenA4	NP_001139603.1	X		X
chickenA5	NP_001039293.1	X		X
chickenA7	NP_001186367.1	X	X	
chimpanzeeA1	XP_001151564.2			X
chimpanzeeA11	XP_522053.2		X	
chimpanzeeA12	XP_001165302.1		X	
chimpanzeeA13	XP_526175.2		X	
chimpanzeeA15	XP_001148217.1			X

chimpanzeeA16	XP_518688.2			X
chimpanzeeA17	XP_004055010.1		X	
chimpanzeeA18	XP_009457982.1		X	
chimpanzeeA2	XP_003311617.1			X
chimpanzeeA20	XP_001169267.2		X	
chimpanzeeA23	XP_009448686.1		X	
chimpanzeeA24	XP_009421581.1		X	
chimpanzeeA3	XP_001152133.1			X
chimpanzeeA31	XP_009429710.1		X	
chimpanzeeA4	XP_001163062.1			X
chimpanzeeA5	XP_003310855.1			X
chimpanzeeA6	XP_001160252.1		X	
chimpanzeeA7	XP_001137440.1		X	
chimpanzeeA8	XP_508510.4		X	
chimpanzeeA9	XP_001160644.1		X	
cionaA15	XP_009860851.1	X		
cionaA16	XP_002124941.1	X		X
cionaOCTN	XP_002132109.1	X		X
coelacanthA17	XP_005994492.1		X	
coelacanthA23	XP_006004499.1		X	
coelacanthA3	XP_006012004.1	X		X
coelacanthA31	XP_006001096.1		X	
cowA1	NP_001094568.1			X
cowA10	XP_002699367.1		X	
cowA14	XP_601004.3		X	
cowA15	NP_001180022.1			X
cowA16	NP_001069792.2			X
cowA17	XP_010807280.1		X	
cowA2	XP_599284.3			X
cowA20	XP_002699386.1		X	
cowA3	XP_002690418.1			X
cowA31	XP_005218549.1		X	
cowA4	NP_001193918.1			X
cowA5	NP_001039967.1			X
cowA6	NP_001001143.1		X	
cowA7	NP_001094517.1		X	
cowA8	NP_001193175.1		X	
cowA9	NP_001039471.1		X	
dogA10	XP_533256.2		X	
dogA11	XP_854924.2		X	
dogA12	NP_001271402.1		X	
dogA13	XP_542706.2		X	
dogA14	XP_005634309.1		X	
dogA15	XP_005630710.1			X
dogA16	XP_532263.2			X
dogA17	XP_005623963.1		X	
dogA2	NP_001273890.1			X

dogA20	XP_854865.2		X	
dogA3	XP_533467.3			X
dogA31	XP_005620617.1		X	
dogA4	XP_005626571.1			X
dogA5	XP_860734.1			X
dogA6	XP_005631653.1		X	
dogA8	XP_005631651.1		X	
dogA9	XP_533255.2		X	
ferretA1	XP_004770900.1			X
flySLC22[1]	NP_651238.2	X		X
flySLC22[2]	NP_524479.1	X		X
frogA15	Q6NUB3.2	X		X
frogA16	AAH80416.1	X		X
frogA2	NP_001087673.1	X		X
frogA4	NP_001088049.1	X		X
frogA5	NP_001080898.1	X		X
frogA6	NP_001087661.1	X	X	
horseA1	XP_001491464.4			X
horseA10	XP_001503029.2		X	
horseA12	XP_001489890.3		X	
horseA13	XP_001488889.1		X	
horseA14	XP_005601107.1		X	
horseA15	XP_001496384.2			X
horseA16	XP_005597022.1			X
horseA2	XP_001500595.2			X
horseA24	XP_008505067.1		X	
horseA4	XP_005599500.1			X
horseA5	XP_001917996.1			X
horseA7	XP_001918258.2		X	
horseA8	XP_001495264.1		X	
horseA9	XP_001495463.3		X	
humanA1	NP_003048.1	X		X
humanA10	NP_001034841.3	X	X	
humanA11	NP_060954.1	X	X	
humanA12	NP_653186.2	X	X	
humanA13	NP_004247.2	X	X	
humanA14	XP_005265641.1	X	X	
humanA15	NP_060890.2	X		X
humanA16	NP_149116.2	X		X
humanA17	NP_057693.3	X	X	
humanA18	XP_006725190.1	X	X	
humanA2	NP_003049.2	X		X
humanA20	A6NK97.1	X	X	
humanA23	AAI28582.1	X	X	
humanA24	Q8N4F4.1	X	X	
humanA25	NP_955384.3	X	X	
humanA3	NP_068812.1	X		X

humanA31	A6NKX4.3	X	X	
humanA4	NP_003050.2	X		X
humanA5	NP_003051.1	X		X
humanA6xB	NP_695008.1	X	X	
humanA7	NP_006663.2	X	X	
humanA8	NP_004245.2	X	X	
humanA9	NP_543142.2	X	X	
lampreyOAT	Jl12567*	X	X	
lampreyOATlike	Jl188*	X	X	
lampreyOATrelated	JL10482*	X	X	
lampreyOCT	Jl2643*	X		X
macaqueA1	XP_005551559.1			X
macaqueA11	XP_001084980.1		X	
macaqueA12	NP_001258575.1		X	
macaqueA13	XP_001087330.1		X	
macaqueA14	XP_001087096.1	X	X	
macaqueA15	XP_001112207.1			X
macaqueA16	XP_001088078.1			X
macaqueA2	XP_005551558.1			X
macaqueA3	XP_005551557.1			X
macaqueA4	XP_005557778.1			X
macaqueA5	XP_002804555.1			X
macaqueA6	NP_001252596.1		X	
macaqueA8	NP_001181622.1		X	
marmosetA1	XP_008993585.1	X		X
marmosetA10	XP_003734338.1	X	X	
marmosetA12	XP_003734362.1	X		
marmosetA13	XP_002759794.1	X	X	
marmosetA15	XP_002751344.2	X		X
marmosetA16	XP_008993154.1	X		X
marmosetA17	XP_002753685.1	X	X	
marmosetA18	XP_009006935.1	X	X	
marmosetA2	XP_008993586.1	X		X
marmosetA20	XP_002755570.2	X	X	
marmosetA23	XP_008992308.1	X	X	
marmosetA3	XP_003732818.1	X		X
marmosetA31	XP_008984632.1	X	X	
marmosetA4	XP_002744655.2	X		X
marmosetA5	XP_008989619.1	X		X
marmosetA6	XP_009006608.1	X		
marmosetA7	XP_002746615.1	X	X	
mouseA1	NP_033228.2	X		X
mouseA12	NP_033229.3	X	X	
mouseA13	NP_598741.2	X	X	
mouseA14	NP_001032838.1	X	X	
mouseA15	NP_001034460.2	X		X
mouseA16	NP_081848.1	X		X

mouseA17	NP_067526.2	X	X	
mouseA18	NP_032793.2	X	X	
mouseA19	NP_659034.1	X	X	
mouseA2	NP_038695.1	X		X
mouseA20	NP_941052.1	X	X	
mouseA21	NP_062697.1	X		X
mouseA22	NP_759010.1	X	X	
mouseA23	AAH53705.1	X	X	
mouseA26	NP_666344.1	X	X	
mouseA27	NP_599017.1	X	X	
mouseA28	XP_006527242.1	X	X	
mouseA29	XP_006527123.1	X	X	
mouseA3	NP_035525.1	X		X
mouseA30	NP_795976.1	X	X	
mouseA4	NP_062661.1	X		X
mouseA5	NP_035526.1	X		X
mouseA6	NP_032792.2	X	X	
mouseA7	NP_659105.2	X	X	
mouseA8	NP_112471.3	X	X	
opossumA1	XP_001371462.2	X		X
opossumA11	XP_001367844.2	X	X	
opossumA12	XP_007506189.1	X	X	
opossumA13	XP_007500655.1	X	X	
opossumA14	XP_007500651.1	X	X	
opossumA15	XP_001364080.2	X		X
opossumA16	XP_007485264.1	X		X
opossumA17	XP_001380044.1	X	X	
opossumA2	XP_001381474.1	X		X
opossumA23	XP_003340725.1	X	X	
opossumA3	XP_001381481.2	X		X
opossumA31	XP_007477376.1	X	X	
opossumA4	XP_001366071.1	X		X
opossumA5	XP_001366136.2	X		X
opossumA6	XP_007497796.1	X	X	
opossumA7	XP_007484011.1	X	X	
orangutanA11	NP_001127182.1		X	
orangutanA2	NP_001126767.1			X
orangutanA24	NP_001125361.1		X	
orangutanA7	NP_001127633.1		X	
orangutanA8	NP_001125961.1		X	
pandaA1	XP_002925382.1			X
pandaA10	XP_002925418.1		X	
pandaA12	XP_002916755.1		X	
pandaA13	XP_002914692.1		X	
pandaA14	XP_002914690.1		X	
pandaA16	XP_002927601.1			X
pandaA2	XP_002925381.1			X

pandaA4	XP_002912999.1			X
pandaA5	XP_002912941.1			X
pandaA7	XP_002914519.1		X	
pandaA9	XP_002925397.1		X	
pigA1	NP_999154.1			X
pigA15	XP_001927939.2			X
pigA16	XP_005652520.1			X
pigA2	NP_999067.1			X
pigA4	NP_001139224.1			X
pigA6	NP_001001261.1		X	
pigA7	NP_001038082.1		X	
pigA8	NP_999620.1		X	
platypusA1	XP_007670399.1	X		X
platypusA13	XP_001521203.2	X	X	
platypusA15	XP_007658941.1	X		X
platypusA23	XP_007667285.1		X	
platypusA24	XP_007667831.1	X	X	
platypusA31	XP_007671059.1	X	X	
platypusA5	XP_007668008.1	X		X
platypusA7	XP_007654505.1	X	X	
pufferfishA15	XP_003963824.1			X
pufferfishA23	XP_003979178.1	X	X	
pufferfishA31	XP_003969973.1	X	X	
rabbitA1	NP_001075491.1			X
rabbitA17	XP_008267601.1		X	
rabbitA2	NP_001075584.1			X
rabbitA24	XP_008272574.1		X	
rabbitA4	NP_001164817.1			X
rabbitA6	NP_001075596.1		X	
rabbitA7	NP_001076111.1		X	
rabbitA8	NP_001075590.1		X	
ratA1	NP_036829.1			X
ratA12	NP_001030115.1		X	
ratA13	NP_001119757.1		X	
ratA14	NP_001101663.1		X	
ratA15	NP_001101177.1			X
ratA17	XP_006252027.1		X	
ratA18	XP_006230837.1		X	
ratA2	Q9R0W2.1			X
ratA22	NP_001013969.1		X	
ratA24	NP_620263.1		X	
ratA3	NP_062103.1			X
ratA4	NP_071606.1			X
ratA5	NP_062142.1			X
ratA6	NP_058920.1		X	
ratA7	NP_445989.2		X	
ratA8	NP_112622.1		X	

salmonA2	ACN11115.1			X
salmonA4	ACN60266.1	X		X
salmonA6	NP_001133616.1		X	
salmonA7	ACI33410.1		X	
seaurchA15	XP_003730263.1	X		X
seaurchSLC22[1]	SPU_006524**	X		X
seaurchSLC22[2]	XP_787726.3	X		X
seaurchSLC22[3]	XP_796008.3	X		X
seaurchSLC22[4]	XP_783608.3	X		X
sharkA15	XP_007889787.1	X		X
sharkA16	XP_007892163.1	X		X
sharkA18	XP_007885570.1	X	X	
sharkA2	sINCAMP00000022649	X		X
sharkA20	XP_007910015.1	X	X	
sharkA23	XP_007900861.1	X	X	
sharkA3	XP_007896508.1	X		X
sharkA31	XP_007887754.1	X	X	
sharkA5	XP_007899456.1	X		X
sharkA7	XP_007890506.1	X	X	
shrewA5	XP_004610019.1			X
tasdevilA8	XP_003774532.1	X	X	
zebrafinchA16	XP_002192085.1			X
zebrafinchA2	XP_002189094.2			X
zebrafinchA7	XP_002195618.1		X	
zebrafishA13	NP_001070840.2	X	X	
zebrafishA15	NP_001103169.1	X		X
zebrafishA16	NP_001020659.1	X		X
zebrafishA17	XP_002666780.2	X	X	
zebrafishA18	NP_001032462.1	X	X	
zebrafishA2	NP_998315.1	X		X
zebrafishA5	NP_957143.1	X		X
zebrafishA6	NP_996960.1	X	X	
zebrafishA7	NP_001077330.1	X	X	
	Sequence Totals	163	175	134
*must use sequence in jlampreygenome database or use their sequence retrival				
**use http://www.echinobase.org/				
