# Supplementary material for: Evolutionary Analysis and Classification of OATs, OCTs, OCTNs, and Other SLC22 Transporters: Structure-Function Implications and Analysis of Sequence Motifs
Source: PLoS One. 2015 Nov 4;10(11):e0140569. doi: 10.1371/journal.pone.0140569 (PMC4633038; doi:10.1371/journal.pone.0140569)
Supplement: S2 Table — (PDF) [file pone.0140569.s008.pdf]

Supplemental Table 2

Evolutionarily Conserved Motif Sequences (green/dark green residues in Figures S4-S6)

Motif Letter*	A	B	C	D	E	F	G	H
Motif Number*	Motif 9	Motif 8	Motif 7	Motif 5	Motif 3	Motif 14	Motif 6	Motif 2
SLC22A15	VEEAFQAVGEMGIYQ			TSIASEWFLIAN	GVISFGQLSDRFGRK	LFAIANGFSPSYEFFAVTRF	GIAQYALLGYFIRSWRTLAI	IPESPRWLYSQGRLSEA
SLC22A16	FEGIYDHVGHFGRFQ	IHYLASVFMGVTPHHVCRPP	EYTGSKKEFPCVDGYIYDQN	STAVTQWNLVCD	GSVTFGYFSDRLGRR	LFGIAAAFAVDYYTFMAARF	GTLLVALTGYLVRTWWLYQM	LPETPFWLLSEGRYEEA
SLC22A17				TNAIGQWDLVCD	GYLFLGYPADRFGRR	PCGVGGAAAGSSTGVMALRF	GHFLFLGLALVSKDWRFLQR	FLESARWLIVKRQIEEA
SLC22A18					GGPVFGRFADQRGAR	VVGLAMAWMSSVFHFCLLVP		
SLC22A23				QNVVSKWDLVCD	GYLITGCIADWVGRR	LTVALSVNVTMFSTLRFFEG	GQFLMPGLAALCRDWQVLQA	FPESLRWLMATQQFESA
SLC22A31				AAAGPTWNLVCG	GCVILGAGCDRFGRR	GLGASEALAASFPTLLVLRL	GTLLLPGLAALVQDWRLLQG	FPESPCWLLATGQVARA
SLC22A6	FNDLLQQVGGVGRFQ	SHNTLQNFTAAIPTHHCRPP	EANGTGATEPCTDGWIYDNS	STIVTEWDLVCS	GAMVFGYLADRLGRR	VSGTCAAFAPNFPIYCAFRL	GQFLLAGVAYAVPHWRHLQL	FIESARWHSSSGRLDLT
SLC22A8	FSEILDRVGSMGHFQ	NHNLLQIFTAATPVHHCRPP	PNDTQRAMEPCLDGWVYNST	DSIVTEWDLVCN	GGLVLGDLSDRFGRR	ASGSGAAFSPTFPIYMVFRF	GQFILPGLAYAIPQWRWLQL	TPESIRWLVLSGKSSKA
SLC22A12	FSELLDLVGGLGRFQ	TQSMLENFSAAVPSHRCWAP	TSWSEADTEPCVDGWVYDRS	STIVAKWNLVCD	GAAACGPASDRFGRR	VMGTAAAFAPAFPVYCLFRF	GHGLTAAVAYGVRDWTLLQL	LAESARWLLTTGRLDWG
SLC22A13	FVQVLAEIGDFGRFQ	FYFFAHVFMVLDEPHHCAVA	LSHRFNETQPCDMGWEYPEN	PSLKNEFNLVCD	GTLMFGPLCDRIGRK	LIGLATAFVPSFELYMALRF	GQMVLAGLAYGFRNWRLLQI	LPESARWLLTRGRMDEA
SLC22A14	FANLLDAVGEFGTFQ		IQFGLNDTDTCQDGWIYPDA	RSLINEFDLVCG	GSLIFRLITDKMGRY	IFGFGTAFMNSFHLYLFFRF	GAVLLTGIAYSLPHWQLLFL	LPESPRWLMMKGKVKEA
SLC22A1	VDDILEQVGESGWFQ	PICVGIVFLGFTPDHHCQSP	TNRSHLPLGPCQDGWVYDTP	SSIVTEFNLVCA	GSLGVGYFADRFGRK	VSGVLMAFSPNYMSMLLFRL	GLVALTGLAYALPHWRWLQL	VPESPRWLLSQKRNTEA
SLC22A2	VDDVLEHGGEFHFFQ	PIYVGIVFLGFTPDHRCRSP	TNRSRLPLGPCRDGWVYETP	SSIVTEFNLVCA	GSMSIGYIADRFGRK	AAGVLMAISPTYTWMLIFRL	GLLVLAGVAYALPHWRWLQF	IPESPRWLISQNKNAEA
SLC22A3	FDEALQRVGEFGRFQ		FPNRSAPLVPCRGGWRYAQA	STIVSEFDLVCV	GAFTLGYAADRYGRI	VTGVVVAFAPNFPVFVIFRF	GIIILPGIAYFIPNWQGIQL	VPESPRWLITRKKGDKA
SLC22A4	YDEVIAFLGEWGPFQ	FNGMSVVFLAGTPEHRCRVP	VDLGQLEQESCLDGWEFSQD	STVVTEWNLVCE	GSFVSGQLSDRFGRK	VLFATMAVQTGFSFLQIFSI	GYMLLPLFAYFIRDWRMLLL	IPESPRWLISQRRFREA
SLC22A5	YDEVTAFLGEWGPFQ	FTGLSSVFLIATPEHRCRVP	VDLGQLEQESCLDGWEFSQD	STIVTEWNLVCE	GSFISGQLSDRFGRK	GFSFLQIFSKNFEMFVVLFV	GYMVLPLFAYFIRDWRMLLV	IPESPRWLISQGRFEEA

Motif Letter*	I	J	K	L	M	N	O	P
Motif Number*	Motif 12	Motif 11	Motif 10	Motif 16	Motif 4	Motif 13	Motif 15	Motif 1
SLC22A15	SFLDLFRYRVLLGHTLILMF	IWFVCSLVYYGLTLS	SIYANLALSGLIEIP	FGRKRTLSAFLCLGG	IVYIYTSELYPTVIRNVGLG	CSMFSRVGGIIAPFIPSLKY	PFIVFGATGLTSGLLSLLLP	ETLNSPLLETFSDLQVYSYR
SLC22A16		IWFTGSLGFYSFSLN	NEYLNLFLLGVVEIP	VGRRTVLAYSLFCSA	LIYLYTAELYPTIVRSLAVG	GSMVCRLASILAPFSVDLSS	PQLFVGTMALLSGVLTLKLP	ETLGKRLATTWEEAAKLESE
SLC22A17	SFASLLNYRNIWKNLLILGF			FGRRGILLLSMTLTG	LSTLLAAEVIPTTVRGRGLG		QHVVLAACALLCILSIMLLP	ETKRKLLPEVLRDGELCRRP
SLC22A18	AIASLLRLPDVPRIFLVKVA							
SLC22A23	AVGSLSVFFCAEITPTVIRC			LGRRGGLLLFMILTA		AVGSLSVFFCAEITPTVIRC		ESRDQNLPENISNGEHYTRQ
SLC22A31	SPLGLLRTRVTWRNGLILGF			CGRRPVLLLGTMVTG	LSSLFAAEVFPTVIRGAGLG		QQVVFASLAVLALLCVLLLP	ESRSRGLPQSLQDADRLRRS
SLC22A6	SAMELLRCPTLRHLFLCLSM	LWFATSFAYYGLVMD	SIYLIQVIFGAVDLP	LGRRPAQMAALLLAG	CIFLYTGELYPTMIRQTGMG	GSTMARVGSIVSPLVSMTAE	PLFIYGAVPVAASAVTVLLP	ETLGQPLPDTVQDLESRKGK
SLC22A8	TASDLFRIPMLRRMTFCLSL	AWFATGFAYYSLAMG	NLYILQIIFGGVDVP	LGRHTTQAAALLLAG	CLFLYTSELYPTVIRQTGMG	SNLWTRVGSMVSPLVKITGE	PNIIYGITALLGGSAALFLP	ETLNQPLPETIEDLENWSLR
SLC22A12	SLGTLLRMPGLRFRTCISTL	CWFAFGFTFFGLALD	NIFLLQMFIGVVDIP	LGRRPTLAASLLLAG	CITIYSSELFPTVLRMTAVG	GQMAARGGAILGPLVRLLGV	PLLVYGTVPVLSGLAALLLP	ETQSLPLPDTIQDVQNQAVK
SLC22A13	NALDLFRHPQLRKVTLIIFC	VWFVDSLGYYGLSLQ	DVYLTQLIFGAVEVP	FGRKWSQLGTLVLGG	ISYVYSAELFPTILRQTGMG	VGIFSRIGGILTPLVILLGE	PMLIYGSLPIVAGLLCTLLP	ETHGQGLKDTLQDLELGPHP
SLC22A14	SVLDFCKNRQLCKVTLVMSC	VWFTVSYTYFTLSLR	SVHFRHVVPSIMEVP	IGRKWSLAVTLLQAI	VFFLYTAELLPTVLRATGLG			ETRDQPLSESLNHSSQIRNK
SLC22A1	SFADLFRTPRLRKRTFILMY	LWFTDSVLYQGLILH	NLYLDFLYSALVEIP	VGRIYPMAMSNLLAG	MICLVNAELYPTFVRNLGVM	CSSLCDIGGIITPFIVFRLR	PLILFAVLGLLAAGVTLLLP	ETKGVALPETMKDAENLGRK
SLC22A2	SFLDLVRTPQIRKHTMILMY	NWFTSSVLYQGLIMH	NIYLDFFYSALVEFP	IGRRYPWAASNMVAG	IVCLVNAELYPTFIRNLGVH	CSSMCDIGGIITPFLVYRLT	PLMVFGVLGLVAGGLVLLLP	ETKGKALPETIEEAENMQRP
SLC22A3	SFLDLVRTPQMRKCTLILMF	AWFTSAVVYQGLVMR	NLYIDFFISGVVELP	LGRRLPFAASNIVAG	IVYLVNSELYPTTLRNFGVS	CSGLCDFGGIIAPFLLFRLA	PLIIFGILASICGGLVMLLP	ETKGIALPETVDDVEKLGSP
SLC22A4	FILDLFRTRNIAIMTIMSLL	LWMLTSVGYFALSLD	DAYLNCFLSALIEIP	LPRRYIIAAVLFWGG	MLYVFTAELYPTLVRNMAVG	TSTASRVGSIIAPYFVYLGA	PYIVMGSLTVLIGILTLFFP	ESLGMTLPETLEQMQKVKWF
SLC22A5	NILDLLRTWNIRMVTIMSIM	LWMTISVGYFGLSLD	DIFVNCFLSAMVEVP	LPRRYSMATALFLGG	MVYVYTAELYPTVVRNMGVG	SSTASRLGSILSPYFVYLGA	PYILMGSLTILTAILTLFLP	ESFGTPLPDTIDQMLRVKGM

Blank Cells: Motif sequences that are not found in that particular sequence.
Green: Only SLC22A6, SLC22A8, SLC22A12, SLC22A1, SLC22A2, SLC22A4, and SLC22A5 are displayed in Supplemental Figures 4-6 (green).
*The motif letter order describes the order in which the motifs appear in the sequence. The motif number describes the order in which MEME identified the motif.
