# Supplementary material for: Evolutionary Analysis and Classification of OATs, OCTs, OCTNs, and Other SLC22 Transporters: Structure-Function Implications and Analysis of Sequence Motifs
Source: PLoS One. 2015 Nov 4;10(11):e0140569. doi: 10.1371/journal.pone.0140569 (PMC4633038; doi:10.1371/journal.pone.0140569)
Supplement: S3 Table — (PDF) [file pone.0140569.s009.pdf]

Supplemental Table 3
Subclade Specific Sequences
(red/blue residues in Figures S4-S6)

Transporter	Region	Sequence	E-Value
SLC22A1	1-3	MPT	1.8e-664
	19-20	KQ	1.8e-664
	54-65	GVAELSQRCGWS	2.1e-696
	87-106	QCRRYEVDWNQSALSCVDPL	4.2e-559
	131-131	G	4.2e-559
	144-152	DSWKLDLFQ	5.5e-705
	209-228	QGLVSKGNWMAGYTLITEFV	2.7e-619
	267-273	AVSLPTF	4.2e-639
	275-281	FLLYYWC	2.3e-806
	299-314	IKIMDHIAQKNGKLPP	1.4e-568
	368-373	MGATSG	1.9e-695
	398-399	DR	4.4e-639
	415-417	AAC	4.4e-639
	435-447	MCVGRMGITIAIQ	1.3e-678
	468-468	V	1.3e-678
	489-494	EVWQAL	2.8e-621
SLC22A2	2-4	PTT	1.8e-664
	20-21	KQ	1.8e-664
	55-66	GVAELSLRCGWS	2.1e-696
	88-107	QCRRYEVDWNQSTFDCVDPL	4.2e-559
	132-132	G	4.2e-559
	145-153	NSWMLDLFQ	5.5e-705
	210-229	QGLVSKAGWLIGYILITEFV	2.7e-619
	268-274	TVSLPNF	4.2e-639
	276-282	FLLYYWC	2.3e-806
	300-315	MRIIKHIAKKNGKSLP	1.4e-568
	369-374	MGLAGD	1.9e-695
	399-400	DR	4.4e-639
	416-418	AAC	4.4e-639
	436-448	SCLGRMGITMAYE	1.3e-678
	469-469	I	1.3e-678
	490-495	NIWLEL	2.8e-621
SLC22A3	1-3	MPS	1.8e-664
	19-20	RR	1.8e-664
	47-66	DHYWCRGPSAAALAERCGWS	2.1e-696
	137-137	H	1.2e-561
	150-158	NAWMLDLTQ	5.5e-705
	215-234	QGVFGKGTWMTCYVIVTEIV	2.7e-619
	273-279	AITLPSF	4.2e-639
	281-287	FLLYYWV	2.3e-806
	305-320	LQILRRIAKCNGKYLS	1.4e-568
	372-377	LGIIGG	1.9e-695

	402-403	ER	4.4e-639
	419-421	VAC	4.4e-639
	439-451	ATLGRLGITMAFE	1.3e-678
	472-472	L	8.5e-782
	493-498	AVWLEL	2.8e-621
SLC22A4	1-3	MRD	1.1e-603
	19-20	RL	1.1e-603
	22-33	FFLLSASIIPNG	2.6e-523
	54-65	DAANLSSAWRNN	2.9e-651
	77-96	VPHSCSRYRLATIANFSALG	1.1e-529
	99-102	PGRD	1.2e-605
	138-145	DNWKVPLT	3.4e-636
	171-171	N	6.8e-589
	205-224	MGQISNYVVAFILGTEILGK	1.7e-599
	239-241	FAV	4.6e-596
	270-276	CVPLWWF	3.5e-640
	294-309	EDIIQKAAKMNNIAVP	3.1e-515
	363-368	APNLHG	4.2e-628
	384-394	AYITAWLLLRT	9.6e-518
	440-442	AFS	8.4e-612
	484-488	YNRML	9.0e-572
SLC22A5	1-3	MRD	1.1e-603
	19-20	RL	1.1e-603
	22-33	FFLLSASIIPNG	2.6e-523
	54-65	DAANLSSAWRNH	2.9e-651
	77-96	VPHSCRRYRLATIANFSALG	1.1e-529
	99-102	PGRD	1.2e-605
	138-145	DDWKAPLT	3.4e-636
	171-180	NVLFVTMGMQ	6.8e-589
	205-224	MGQISNYVAAFVLGTEILGK	1.7e-599
	239-241	YAF	4.6e-596
	270-276	CVALWWF	3.5e-640
	294-309	EVIIRKAAKANGIVVP	3.1e-515
	365-370	TPNLHG	4.2e-628
	386-396	AYVLAWLLLQY	9.6e-518
	442-444	AFS	8.4e-612
	486-490	YDRFL	9.0e-572
SLC22A6	67-86	LPRDRQGQPESCLRFTSPQW	1.0e-1078
	115-117	TFP	3.2e-1323
	130-142	HRALRQLAQSLYM	1.1e-1079
	194-196	LSG	1.2e-878
	201-220	GISLNCMTLNVEWMPIHTRA	1.6e-636
	253-259	LVSAPFF	7.3e-977
	261-267	FFIYSWF	9.6e-751
	285-298	LRALQRVARINGKR	4.6e-894

	320-324	GKGQA	2.1e-638
	360-365	LQGFGV	7.6e-806
	414-433	VIPQDQSIVRTSLAVLGKGC	5.0e-719
SLC22A8	62-81	LPMGPNGKPERCLRFVHPPN	1.0e-1078
	105-105	K	3.2e-1323
	118-130	SNKLKEMAQSIFM	1.1e-1079
	182-184	LCG	1.2e-878
	189-208	GITLSTVILNVEWVPTRMRA	1.6e-636
	241-247	TVSIPFF	7.3e-977
	249-255	FFLSSWW	9.6e-751
	273-286	LKILRRVAVFNGKK	4.6e-894
	308-312	AKAKY	2.1e-638
	348-353	VEEFGV	7.6e-806
	402-421	FVPLDLQTVRTVLAVFGKGC	5.0e-719
SLC22A12	77-96	IPPGPNQRPHQCRRFRQPQW	1.0e-1078
	126-128	IFT	3.2e-1323
	141-153	SHALKPMAQSIYL	1.1e-1079
	205-207	LLA	1.2e-878
	212-231	GVMMNTGTLLMEWTAARARP	1.6e-636
	264-270	VVSVPFF	7.3e-977
	272-278	CFLYSWW	9.6e-751
	296-309	LQELWRVAAINGKG	4.6e-894
	331-335	GQPPA	2.1e-638
	371-376	LQALGS	7.6e-806
	425-444	LVPHEMGALRSALAVLGLGG	5.0e-719
SLC22A13	1-3	MAQ	1.7E-190
	19-20	IQ	1.7E-190
	27-33	VLNFLSP	4.1E-157
	54-67	WVKNHTFNLSAAEQ	9.3E-241
	73-90	PLDTAGHPEPCLMFRPPP	8.7E-175
	119-119	R	7.2E-241
	133-150	RKHLKDTTQSVFMAGLLV	5.1E-234/6.30E-197
	197-201	AVATA	3.9E-206
	214-233	TEWVGPSWRTQAVVLAQCNF	2E-206
	269-270	WA	2.8E-252
	288-288	I	2.8E-252
	292-311	QKAASVNRRKLSPELMNQLV	4.6E-172
	358-360	FGL	2.7E-225
	376-377	AR	2.7E-225
	385-386	QR	1.3E-163
	402-404	LMC	1.3E-163
	455-455	L	3.1E-224
SLC22A14	53-68	ANLLDAVGEFGTFQQR	1.7E-190
	75-94	IPSIMSAFFMFADHFVFTAQ	4.1E-157

	96-105	PYCNTSWILA	9.3E-241
	126-128	GSF	8.7E-175
	146-158	QFGLNDTDTCQDG	7.2E-241
	174-184	DLVCGMETKKD	5.1E-234
	205-210	TDKMGR	6.3E-197
	228-243	TAFMNSFHLYLFFRFG	3.9E-206
	264-278	VGEHRAHAIILGHCF	2E-206
	296-301	WQLLFL	4.7E-222
	315-334	WILPESPRWLMMKGKVKEAK	2.8E-252
	371-376	FCKNRQ	4.3E-228
	418-424	IMEVPAR	2.7E-225
	432-450	QQIGRKWSLAVTLLQAIIW	1.3E-163
SCL22A15	107-119	RSYKVSAASSFFF	4.7e-302
	140-143	KVYL	6.3e-280
	171-171	L	1.6e-312
	178-197	GMSLVAFVLLNECVGTAYWA	1.6e-380
	206-209	FFAV	2.8e-412
	262-262	E	1.1e-412
	293-293	G	3.0e-249
	332-334	LGG	4.5e-403
	350-351	SY	4.5e-403
	361-362	KW	1.5e-309
	378-380	LAC	1.5e-309
	382-401	IVMFLPEKKDTGVFAVVNSH	1.7e-201
	413-416	AAFN	2.4e-489
	437-437	T	1.5e-399
	533-547	EEEFYDADEETQMIK	2.3e-212
SLC22A16	29-35	FQNISCG	7.9e-205
	92-106	NGEIWELSRCSRNKR	2.3e-212
	150-162	RKWLAMLIQPLFM	4.7e-302
	183-186	VVLW	6.3e-280
	214-214	F	1.6e-312
	221-240	GYLVVGFVYVMEFIGMKSRT	2.8e-412
	249-252	FFAV	2.8e-412
	305-305	Q	1.1e-412
	344-364	HNLSYLFYNWSITKRTLTVWL	3.0e-249
	383-385	LGG	4.5e-403
	401-402	AY	4.5e-403
	410-411	DK	1.5e-309
	427-429	LAC	1.5e-309
	456-459	AAFG	2.4e-489
	480-480	S	1.5e-399
SLC22A17	96-104	LGWQVILEQ	
	129-139	GIVLLTLGLVG	
	160-163	LLGF	

	166-185	AGVDLGVYLMRLELCDPTQR	
	198-198	G	
	269-288	MLGEEAQEALQDLENTCPLP	
	314-324	TNFIAHAIRHC	
	334-361	PSDFYLCSLLASGTAALACVFLGVTVDR	
	387-406	DYLNEAAITTFSVLGLFSSQ	
	431-445	LIMALGALGGLSGPA	
	449-456	HMGHGAFL	
	500-519	RQPPPTRCDHVPLLATPNPA	
SLC22A18	148-167	SALGRSSVILLTYVLAATEL	
	203-204	LL	
	220-222	AAL	
SLC22A23	224-233	DNAWKVHIAK	
	241-242	IF	
	258-260	PVL	
	292-292	F	
	295-314	AGIILTLYALRIELCPPGKR	
	327-327	A	
	437-447	NSLTGYGIHHC	
	462-476	LENFYADYYTTASIA	
	488-490	RFL	
	506-507	AS	
	530-549	DSVKDKFSIAFSIVGMFASH	
	570-588	GGLGLVLASAGFGMLTAPI	
	592-611	HNQKGYFLHHIIFACCTLIC	
	615-618	ILLL	
SLC22A31	125-133	DGWKVPLEQ	
	141-142	LL	
	158-160	AVF	
	189-192	LHGG	
	195-214	AGALLALYLARLELCDPPHR	
	227-227	V	
	347-357	SSLVGGGIRAS	
	366-380	VPTFYLPYFLEAGLE	
	392-393	DC	
	409-411	LAS	
	419-438	QYLPGWTVLFLSVLGLLASR	
	463-477	LVLGAGFLGQAAGPL	
	481-488	HGRQGFFL	
	530-549	LLRGRPRQDHLPLLPPSNSY	
NOTE: E-values for SLC22A17, A18, A23 and A31 are not shown as the motifs are not highly conserved across the members.			
