# Supplementary material for: Evolutionary Analysis and Classification of OATs, OCTs, OCTNs, and Other SLC22 Transporters: Structure-Function Implications and Analysis of Sequence Motifs
Source: PLoS One. 2015 Nov 4;10(11):e0140569. doi: 10.1371/journal.pone.0140569 (PMC4633038; doi:10.1371/journal.pone.0140569)
Supplement: S4 Table — (PDF) [file pone.0140569.s010.pdf]

Supplemental Table 4
Specific Residue Information Mapped on Supplemental Figures 4-6

Mapped Transporter	Residue	Position	Change	Substantial Effect on Transport Function		Reference	PMID	Additional Notes
				Yes	No			
hOAT1	C	440	A	+		Astorga et al. 2011	21543413	
	R	454	Q	+		Fujita et al. 2005	15864112	
	R	50	H		+	Fujita et al. 2005	15864112	
	P	104	L		+	Fujita et al. 2005	15864112	
	I	226	T		+	Fujita et al. 2005	15864112	
	A	256	V		+	Fujita et al. 2005	15864112	
	R	293	W		+	Fujita et al. 2005	15864112	
	K	431	A	+		Perry et al. 2006	17038320	
	F	438	A	+		Perry et al. 2006	17038320	
	R	466	K	+		Rizwan et al. 2007	17353191	
	K	394*	A	+		Rizwan et al. 2007	17353191	*Mutant in flOAT1
	R	478*	D	+		Rizwan et al. 2007	17353191	*Mutant in flOAT1
	N	39	Q	+		Tanaka et al. 2004a	14749323	
	N	92***	Q	+		Tanaka et al. 2004a	14749323	***quadruple mutant
	N	56***	Q	+		Tanaka et al. 2004a	14749323	***quadruple mutant
	N	86***	Q	+		Tanaka et al. 2004a	14749323	***quadruple mutant
	N	113***	Q	+		Tanaka et al. 2004a	14749323	***quadruple mutant
	C	49	A	+		Tanaka et al. 2004b	14979872	
	C	335**	A	+		Tanaka et al. 2004b	14979872	**Mutant in mOAT1, quad mutant
	C	379**	A	+		Tanaka et al. 2004b	14979872	**Mutant in mOAT1, quad mutant
	C	427**	A	+		Tanaka et al. 2004b	14979872	**Mutant in mOAT1, quad mutant
	C	434**	A	+		Tanaka et al. 2004b	14979872	**Mutant in mOAT1, quad mutant
	C	122	A		+	Tanaka et al. 2004b	14979872	
	C	172	A		+	Tanaka et al. 2004b	14979872	

	C	183	A		+	Tanaka et al. 2004b	14979872	
	C	200	A		+	Tanaka et al. 2004b	14979872	
	C	78*	A		+	Tanaka et al. 2004b	14979872	*Mutant in mOAT1
	C	99*	A		+	Tanaka et al. 2004b	14979872	*Mutant in mOAT1
	C	326**	A		+	Tanaka et al. 2004b	14979872	**Mutant in mOAT1, quad mutant
hOAT3	C	428	A	+		Astorga et. Al 2011	21543413	
	R	149	S	+		Erdman et. Al 2005	16291576	
	Q	239	STOP	+		Erdman et. Al 2005	16291576	
	I	260	R	+		Erdman et. Al 2005	16291576	
	R	277	Y	+		Erdman et. Al 2005	16291576	
	I	305	F	+		Erdman et. Al 2005	16291576	
	A	310	V		+	Erdman et. Al 2005	16291576	
	A	399	S		+	Erdman et. Al 2005	16291576	
	V	448	I		+	Erdman et. Al 2005	16291576	
	K	370*	A	+		Feng et al. 2001	11331016	*double mutant K370A/R454D
	R	454*	D	+		Feng et al. 2001	11331016	*double mutant K370A/R454D
	K	370*	A	+		Rizwan et al. 2007	17353191	*double mutant K370A/R454D
	R	454*	D,N	+		Rizwan et al. 2007	17353191	*double mutant K370A/R454D
hOCT1	Q	97	K	+		Chen L et al. 2010	20639304	
	P	117	L	+		Chen L et al. 2010	20639304	
	R	206	C	+		Chen L et al. 2010	20639304	
	D	475	E	+		Gorboulev et al. 1999	10570053	Mutant in rOCT1
	A	443	I	+		Gorboulev et al. 2005	15662045	Mutant in rOCT1
	L	447	Y	+		Gorboulev et al. 2005	15662045	Mutant in rOCT1
	Q	448	E	+		Gorboulev et al. 2005	15662045	Mutant in rOCT1
	R	61	C	+		Kerb et al. 2002	12439218	
	C	88	R	+		Kerb et al. 2002	12439218	
	G	401	S	+		Kerb et al. 2002	12439218	
	P	283	L	+		Sakata et al. 2004	14697261	
	P	287	G	+		Sakata et al. 2004	14697261	

	P	341	L	+		Sakata et al. 2004	14697261	
	F	160	L		+	Sakata et al. 2004	14697261	
	R	61	C	+		Shu et al. 2003	12719534	
	L	160	F	+		Shu et al. 2003	12719534	
	G	220	V	+		Shu et al. 2003	12719534	
	P	341	L	+		Shu et al. 2003	12719534	
	G	401	S	+		Shu et al. 2003	12719534	
	G	465	R	+		Shu et al. 2003	12719534	
	S	14	F	+		Shu et al. 2003	12719534	
	L	85	F		+	Shu et al. 2003	12719534	
	S	189	L		+	Shu et al. 2003	12719534	
	R	342	H		+	Shu et al. 2003	12719534	
	M	408	V		+	Shu et al. 2003	12719534	
	M	420	DEL		+	Shu et al. 2003	12719534	
	M	440	I		+	Shu et al. 2003	12719534	
	V	461	I		+	Shu et al. 2003	12719534	
	R	488	M		+	Shu et al. 2003	12719534	
	R	61	C	+		Shu et al. 2007	17476361	
	S	189	L	+		Shu et al. 2007	17476361	
	G	220	V	+		Shu et al. 2007	17476361	
	G	401	S	+		Shu et al. 2007	17476361	
	M	420	DEL	+		Shu et al. 2007	17476361	
	G	465	R	+		Shu et al. 2007	17476361	
	S	14	F	+		Shu et al. 2007	17476361	
	L	160	F		+	Shu et al. 2007	17476361	
	P	341	L		+	Shu et al. 2007	17476361	
	R	342	H		+	Shu et al. 2007	17476361	
	V	408	M		+	Shu et al. 2007	17476361	
	R	488	M		+	Shu et al. 2007	17476361	
	C	322	A	+		Sturm et al. 2007	17567940	Mutant in rOCT1

	C	451	A	+		Sturm et al. 2007	17567940	Mutant in rOCT1
hOCT2	A	270	S	+		Chen Y et al. 2009	19483665	
	Y	447	L	+		Gorboulev et al. 2005	15662045	Mutant in rOCT2
	Q	448	E	+		Gorboulev et al. 2005	15662045	Mutant in rOCT2
	M	165	I	+		Leabman et al. 2002	12142729	metformin, phenformin, procainamide, quinidine
	A	270	S	+		Leabman et al. 2002	12142729	metformin, phenformin, procainamide, quinidine
	R	400	C	+		Leabman et al. 2002	12142729	metformin, phenformin, procainamide, quinidine
	K	432	Q	+		Leabman et al. 2002	12142729	metformin, phenformin, procainamide, quinidine
	A	270	S	+		More et al. 2010	20371711	
	C	51	A	+		Pelis et al. 2012	22573376	
	C	63	A	+		Pelis et al. 2012	22573376	
	C	89	A	+		Pelis et al. 2012	22573376	
	C	103	A	+		Pelis et al. 2012	22573376	
	C	143	A	+		Pelis et al. 2012	22573376	
	C	474	A	+		Pelis et al. 2012	22573376	
	T	199	I	+		Song et al. 2008	18401339	
	T	201	M	+		Song et al. 2008	18401339	
	C	451	A	+		Sturm et al. 2007	17567940	Mutant in rOCT2
	E	447	K,R	+		Zhang et al. 2005	16087669	Mutant in rbOCT2
	E	447	L	+		Zhang et al. 2005	16087669	Mutant in rbOCT2
	N	353*	L	+		Zhang et al. 2005	16087669	Mutant in rbOCT2,*double mutant decreased affinity
	R	403*	I	+		Zhang et al. 2005	16087669	Mutant in rbOCT2,*double mutant decreased affinity
hOCTN2	R	341	A	+		Amat di San Filippo et al. 2003	14506273	
	L	409	W	+		Amat di San Filippo et al. 2003	14506273	
	T	429	I	+		Amat di San Filippo et al. 2003	14506273	
	M	179	L	+		Koizumi et al. 1999	10545605	
	W	283	C	+		Koizumi et al. 1999	10545605	
	S	467	C	+		Koizumi et al. 1999	10545605	
	W	283	R	+		Mayatepek et al. 2000	10612840	
	V	446	F	+		Mayatepek et al. 2000	10612840	

	L	505	F	+		Peltekova et al. 2004	15107849	mapped from hOCTN1
	L	505	F	+		Pochini et al. 2012	22206629	mapped from hOCTN1
	P	478	L	+		Tang et al. 1999	10072434	
	D	165	G	+		Urban et al. 2007	17700366	mapped from hOCTN1
	E	452	K	+		Wang et al. 2000a	10679939	
	R	169	W	+		Wang et al. 2000b	11058897	
	G	242	V	+		Wang et al. 2000b	11058897	
	A	301	D	+		Wang et al. 2000b	11058897	
	W	351	R	+		Wang et al. 2000b	11058897	
	E	452	Q,D,A,K	+		Wang et al. 2000c	10783384	
hURAT1	R	406	C	+		Dinour et al. 2011	21148271	
	G	444	R	+		Dinour et al. 2011	21148271	
	T	217	M	+		Enomoto et al. 2002	12024214	
	E	298	D	+		Enomoto et al. 2002	12024214	
	I	75	T	+		Tasic et al. 2011	22194875	
	R	347	S	+		Tasic et al. 2011	22194875	
	R	434	C	+		Tasic et al. 2011	22194875	
	R	434	H	+		Tasic et al. 2011	22194875	
	V	388	M		+	Tasic et al. 2011	22194875	
