# Supplementary material for: Evolutionary Analysis and Classification of OATs, OCTs, OCTNs, and Other SLC22 Transporters: Structure-Function Implications and Analysis of Sequence Motifs
Source: PLoS One. 2015 Nov 4;10(11):e0140569. doi: 10.1371/journal.pone.0140569 (PMC4633038; doi:10.1371/journal.pone.0140569)
Supplement: S5 Table — (PDF) [file pone.0140569.s011.pdf]

Supplemental Table 5
List of Sequences Used in MEME Input for Family and Subclade-Specific Analyses

Sequence Name	Accession Number	All 6 subclades	Oat	Oat-like	Oat-related	Oct	Octn	Oct-related
amazonmolly15	XP_007568088.1					X		
baboonA1	XP_003898408.1					X		
baboonA11	XP_003909652.1		X					
baboonA12	XP_003909651.1		X					
baboonA15	XP_003892505.1							X
baboonA2	XP_003898410.1					X		
baboonA23	XP_003897025.1				X			
baboonA31	XP_003917369.1				X			
baboonA4	NP_001162226.1						X	
baboonA5	NP_001162227.1						X	
baboonA6	XP_003909713.1		X					
batA17	XP_006776882.1				X			
batA31	XP_005862737.1				X			
batA4	ACC68852.1						X	
bonoboA16	XP_008976203.1							X
bonoboA17	XP_003808141.1				X			
bonoboA18	XP_003816095.1				X			
bonoboA20	XP_003828666.2		X					
bonoboA25	XP_003828572.1		X					
bonoboA3	XP_003807452.1					X		
bonoboA8	XP_003828567.1		X					
bonoboA9	XP_003828570.1		X					
bushbabyA11	ACH92547.1		X					
callicebusA5	ACB21281.1						X	
catA20	XP_003993638.1		X					
catA23	XP_006931564.1				X			
catA31	XP_004001564.1				X			
chickenA13	XP_418529.3	X		X				
chickenA15	XP_416558.2	X						X
chickenA16	XP_419787.3	X						X
chickenA18	XP_421021.3	X			X			
chickenA2	XP_419622.2	X				X		
chickenA23	XP_418968.3	X			X			
chickenA3	XP_419620.4	X				X		
chickenA4	NP_001139603.1	X					X	
chickenA5	NP_001039293.1	X					X	
chickenA7	NP_001186367.1	X						
chimpanzeeA1	XP_001151564.2					X		
chimpanzeeA11	XP_522053.2		X					
chimpanzeeA12	XP_001165302.1		X					
chimpanzeeA13	XP_526175.2			X				
chimpanzeeA15	XP_001148217.1							X
chimpanzeeA16	XP_518688.2							X
chimpanzeeA17	XP_001162356.1				X			
chimpanzeeA18	XP_009457982.1				X			

chimpanzeeA2	XP_003311617.1					X		
chimpanzeeA20	XP_001169267.2		X					
chimpanzeeA23	XP_009448686.1				X			
chimpanzeeA24	XP_009421581.1		X					
chimpanzeeA3	XP_001152133.1					X		
chimpanzeeA31	XP_009429710.1				X			
chimpanzeeA4	XP_001163062.1						X	
chimpanzeeA5	XP_003310855.1						X	
chimpanzeeA6	XP_001160252.1		X					
chimpanzeeA7	XP_001137440.1		X					
chimpanzeeA8	XP_508510.4		X					
chimpanzeeA9	XP_001160644.1		X					
coelacanthA17	XP_005994492.1				X			
coelacanthA23	XP_006004499.1				X			
coelacanthA3	XP_006012004.1	X				X		
coelacanthA31	XP_006001096.1				X			
cowA1	NP_001094568.1					X		
cowA10	XP_002699367.1		X					
cowA14	XP_601004.3			X				
cowA15	NP_001180022.1							X
cowA16	NP_001069792.2							X
cowA17	NP_001124227.1				X			
cowA2	XP_599284.3					X		
cowA20	XP_002699386.1		X					
cowA3	XP_002690418.1					X		
cowA31	XP_005218549.1				X			
cowA4	NP_001193918.1						X	
cowA5	NP_001039967.1						X	
cowA6	NP_001001143.1		X					
cowA7	NP_001094517.1		X					
cowA8	NP_001193175.1		X					
cowA9	NP_001039471.1		X					
dogA10	XP_533256.2		X					
dogA11	XP_854924.2		X					
dogA12	NP_001271402.1		X					
dogA13	XP_542706.2			X				
dogA14	XP_005634309.1			X				
dogA15	XP_005630710.1							X
dogA16	XP_532263.2							X
dogA17	XP_005623963.1				X			
dogA2	NP_001273890.1					X		
dogA20	XP_854865.2		X					
dogA3	XP_533467.3					X		
dogA31	XP_005620617.1				X			
dogA4	XP_005626571.1						X	
dogA5	XP_860734.1						X	
dogA6	XP_005631653.1		X					
dogA8	XP_005631651.1		X					
dogA9	XP_533255.2		X					

ferretA1	XP_004770900.1					X		
flounderA6	O57379.1		X					
flyinglemurA14	XP_008565394.1			X				
frogA15	Q6NUB3.2	X						X
frogA16	AAH80416.1	X						X
frogA2	NP_001087673.1	X				X		
frogA4	NP_001088049.1	X					X	
frogA5	NP_001080898.1	X					X	
frogA6	NP_001087661.1	X						
horseA1	XP_001491464.4					X		
horseA10	XP_001503029.2		X					
horseA12	XP_001489890.3		X					
horseA13	XP_001488889.1			X				
horseA14	XP_005601107.1			X				
horseA15	XP_001496384.2							X
horseA16	XP_005597022.1							X
horseA2	XP_001500595.2					X		
horseA24	XP_008505067.1		X					
horseA4	XP_005599500.1						X	
horseA5	XP_001917996.1						X	
horseA7	XP_001918258.2		X					
horseA8	XP_001495264.1		X					
horseA9	XP_001495463.3		X					
humanA1	NP_003048.1	X				X		
humanA10	NP_001034841.3	X	X					
humanA11	NP_060954.1	X	X					
humanA12	NP_653186.2	X	X					
humanA13	NP_004247.2	X		X				
humanA14	XP_005265641.1	X		X				
humanA15	NP_060890.2	X						X
humanA16	NP_149116.2	X						X
humanA17	NP_057693.3	X			X			
humanA18	XP_006725190.1	X			X			
humanA2	NP_003049.2	X				X		
humanA20	A6NK97.1	X	X					
humanA23	NP_056297.1	X			X			
humanA24	Q8N4F4.1	X	X					
humanA25	NP_955384.3	X	X					
humanA3	NP_068812.1	X				X		
humanA31	A6NKX4.3	X			X			
humanA4	NP_003050.2	X					X	
humanA5	NP_003051.1	X					X	
humanA6	NP_695008.1	X	X					
humanA7	NP_006663.2	X	X					
humanA8	NP_004245.2	X	X					
humanA9	NP_543142.2	X	X					
lampreyA31	JL10482				X			
lampreyOAT	JI12567	X						
lampreyOATlike	JI188	X		X				

lampreyOCT	JI2643	X				X		
macaqueA1	XP_005551559.1					X		
macaqueA11	XP_001084980.1		X					
macaqueA12	NP_001258575.1		X					
macaqueA13	XP_001087330.1			X				
macaqueA14	XP_001087096.1			X				
macaqueA15	XP_001112207.1							X
macaqueA16	XP_001088078.1							X
macaqueA2	XP_005551558.1					X		
macaqueA3	XP_005551557.1					X		
macaqueA4	XP_005557778.1						X	
macaqueA5	XP_002804555.1						X	
macaqueA6	NP_001252596.1		X					
macaqueA8	NP_001181622.1		X					
marmosetA1	XP_008993585.1					X		
marmosetA10	XP_003734338.1		X					
marmosetA15	XP_002751344.2							X
marmosetA16	XP_008993154.1							X
marmosetA17	XP_002753685.1				X			
marmosetA18	XP_009006935.1				X			
marmosetA2	XP_008993586.1					X		
marmosetA20	XP_002755570.2		X					
marmosetA23	XP_008992308.1				X			
marmosetA3	XP_003732818.1					X		
marmosetA31	XP_008984632.1				X			
marmosetA4	XP_002744655.2						X	
marmosetA5	XP_008989619.1						X	
marmosetA7	XP_002746615.1		X					
mouseA1	NP_033228.2	X				X		
mouseA12	NP_033229.3	X	X					
mouseA13	NP_598741.2	X		X				
mouseA14	NP_001032838.1	X		X				
mouseA15	NP_001034460.2	X						X
mouseA16	NP_081848.1	X						X
mouseA17	NP_067526.2	X			X			
mouseA18	NP_032793.2	X			X			
mouseA19	NP_659034.1	X	X					
mouseA2	NP_038695.1	X				X		
mouseA20	NP_941052.1	X	X					
mouseA21	NP_062697.1	X					X	
mouseA22	NP_759010.1	X	X					
mouseA23	AAH53705.1	X			X			
mouseA26	NP_666344.1	X	X					
mouseA27	NP_599017.1	X	X					
mouseA28	XP_006527242.1	X	X					
mouseA29	XP_006527123.1	X	X					
mouseA3	NP_035525.1	X				X		
mouseA30	NP_795976.1	X	X					
mouseA4	NP_062661.1	X					X	

mouseA5	NP_035526.1	X					X	
mouseA6	NP_032792.2	X	X					
mouseA7	NP_659105.2	X	X					
mouseA8	NP_112471.3	X	X					
opossumA1	XP_001371462.2	X				X		
opossumA11	XP_001367844.2	X						
opossumA12	XP_007506189.1	X						
opossumA13	XP_007500655.1	X		X				
opossumA14	XP_007500651.1	X						
opossumA15	XP_001364080.2	X						X
opossumA16	XP_007485264.1	X						X
opossumA17	XP_001380044.1	X			X			
opossumA2	XP_001381474.1	X				X		
opossumA23	XP_003340725.1				X			
opossumA3	XP_001381481.2	X				X		
opossumA31	XP_007477376.1	X			X			
opossumA4	XP_001366071.1	X					X	
opossumA5	XP_001366136.2	X					X	
opossumA6	XP_007497796.1	X						
opossumA7	XP_007484011.1	X						
orangutanA11	NP_001127182.1		X					
orangutanA2	NP_001126767.1					X		
orangutanA24	NP_001125361.1		X					
orangutanA7	NP_001127633.1		X					
orangutanA8	NP_001125961.1		X					
pandaA1	XP_002925382.1					X		
pandaA10	XP_002925418.1		X					
pandaA12	XP_002916755.1		X					
pandaA13	XP_002914692.1			X				
pandaA14	XP_002914690.1			X				
pandaA16	XP_002927601.1							X
pandaA2	XP_002925381.1					X		
pandaA4	XP_002912999.1						X	
pandaA5	XP_002912941.1						X	
pandaA7	XP_002914519.1		X					
pandaA9	XP_002925397.1		X					
pigA1	NP_999154.1					X		
pigA15	XP_001927939.2							X
pigA16	XP_005652520.1							X
pigA2	NP_999067.1					X		
pigA4	NP_001139224.1						X	
pigA6	NP_001001261.1		X					
pigA7	NP_001038082.1		X					
pigA8	NP_999620.1		X					
platypusA1	XP_007670399.1	X				X		
platypusA13	XP_001521203.2	X		X				
platypusA15	XP_007658941.1	X						X
platypusA23	XP_007667285.1				X			
platypusA24	XP_007667831.1	X	X					

platypusA31	XP_007671059.1	X			X			
platypusA5	XP_007668008.1	X					X	
platypusA7	XP_007654505.1	X						
pufferfishA15	XP_003963824.1							X
pufferfishA23	XP_003979178.1	X			X			
pufferfishA31	XP_003969973.1	X			X			
rabbitA1	NP_001075491.1					X		
rabbitA17	XP_008267601.1				X			
rabbitA2	NP_001075584.1					X		
rabbitA24	XP_008272574.1		X					
rabbitA4	NP_001164817.1						X	
rabbitA6	NP_001075596.1		X					
rabbitA7	NP_001076111.1		X					
rabbitA8	NP_001075590.1		X					
ratA1	NP_036829.1					X		
ratA12	NP_001030115.1		X					
ratA13	NP_001119757.1			X				
ratA14	NP_001101663.1			X				
ratA15	NP_001101177.1							X
ratA17	NP_803156.2				X			
ratA18	NP_001004260.1				X			
ratA2	Q9R0W2.1					X		
ratA22	NP_001013969.1		X					
ratA24	NP_620263.1		X					
ratA3	NP_062103.1					X		
ratA4	NP_071606.1						X	
ratA5	NP_062142.1						X	
ratA6	NP_058920.1		X					
ratA7	NP_445989.2		X					
ratA8	NP_112622.1		X					
salmonA2	ACN11115.1					X		
salmonA4	ACN60266.1	X					X	
sharkA15	XP_007889787.1	X						X
sharkA16	XP_007892163.1	X						X
sharkA18	XP_007885570.1	X			X			
sharkA2	sINCAMP00000022649	X				X		
sharkA20	XP_007910015.1	X						
sharkA23	XP_007900861.1	X			X			
sharkA3	XP_007896508.1					X		
sharkA31	XP_007887754.1	X			X			
sharkA5	XP_007899456.1	X					X	
sharkA7	XP_007890506.1	X						
shrewA5	XP_004610019.1						X	
zebrafinchA16	XP_002192085.1							X
zebrafinchA2	XP_002189094.2					X		
zebrafishA13	NP_001070840.2	X		X				
zebrafishA15	NP_001103169.1	X						X
zebrafishA16	NP_001020659.1	X						X
zebrafishA17	XP_002666780.2	X			X			

zebrafishA18	NP_001032462.1	X			X			
zebrafishA2	NP_998315.1	X				X		
zebrafishA5	NP_957143.1	X					X	
zebrafishA6	NP_996960.1	X						
zebrafishA7	NP_001077330.1	X						
	Total Sequences	111	88	22	48	51	38	35
